# Association between myosteatosis and impaired glucose metabolism: A deep learning whole‐body magnetic resonance imaging population phenotyping approach

**DOI:** 10.1002/jcsm.13527

**Published:** 2024-07-15

**Authors:** Matthias Jung, Hanna Rieder, Marco Reisert, Susanne Rospleszcz, Johanna Nattenmueller, Annette Peters, Christopher L. Schlett, Fabian Bamberg, Jakob Weiss

**Affiliations:** ^1^ Department of Diagnostic and Interventional Radiology, University Medical Center Freiburg, Faculty of Medicine University of Freiburg Freiburg Germany; ^2^ Division of Medical Physics, Department of Diagnostic and Interventional Radiology, University Medical Center Freiburg, Faculty of Medicine University of Freiburg Freiburg Germany; ^3^ Department of Stereotactic and Functional Neurosurgery, University Medical Center Freiburg, Faculty of Medicine University of Freiburg Freiburg Germany; ^4^ Institute of Epidemiology Helmholtz Zentrum München, German Research Center for Environmental Health Neuherberg Germany; ^5^ Department of Epidemiology, Institute for Medical Information Processing, Biometry and Epidemiology Ludwig Maximilian University of Munich Munich Germany; ^6^ German Center for Diabetes Research (DZD) Neuherberg Germany

**Keywords:** body composition, diabetes, myosteatosis, quantitative MRI, skeletal muscle

## Abstract

**Background:**

There is increasing evidence that myosteatosis, which is currently not assessed in clinical routine, plays an important role in risk estimation in individuals with impaired glucose metabolism, as it is associated with the progression of insulin resistance. With advances in artificial intelligence, automated and accurate algorithms have become feasible to fill this gap.

**Methods:**

In this retrospective study, we developed and tested a fully automated deep learning model using data from two prospective cohort studies (German National Cohort [NAKO] and Cooperative Health Research in the Region of Augsburg [KORA]) to quantify myosteatosis on whole‐body T1‐weighted Dixon magnetic resonance imaging as (1) intramuscular adipose tissue (IMAT; the current standard) and (2) quantitative skeletal muscle (SM) fat fraction (SMFF). Subsequently, we investigated the two measures for their discrimination of and association with impaired glucose metabolism beyond baseline demographics (age, sex and body mass index [BMI]) and cardiometabolic risk factors (lipid panel, systolic blood pressure, smoking status and alcohol consumption) in asymptomatic individuals from the KORA study. Impaired glucose metabolism was defined as impaired fasting glucose or impaired glucose tolerance (140–200 mg/dL) or prevalent diabetes mellitus.

**Results:**

Model performance was high, with Dice coefficients of ≥0.81 for IMAT and ≥0.91 for SM in the internal (NAKO) and external (KORA) testing sets. In the target population (380 KORA participants: mean age of 53.6 ± 9.2 years, BMI of 28.2 ± 4.9 kg/m^2^, 57.4% male), individuals with impaired glucose metabolism (*n* = 146; 38.4%) were older and more likely men and showed a higher cardiometabolic risk profile, higher IMAT (4.5 ± 2.2% vs. 3.9 ± 1.7%) and higher SMFF (22.0 ± 4.7% vs. 18.9 ± 3.9%) compared to normoglycaemic controls (all *P* ≤ 0.005). SMFF showed better discrimination for impaired glucose metabolism than IMAT (area under the receiver operating characteristic curve [AUC] 0.693 vs. 0.582, 95% confidence interval [CI] [0.06–0.16]; *P* < 0.001) but was not significantly different from BMI (AUC 0.733 vs. 0.693, 95% CI [−0.09 to 0.01]; *P* = 0.15). In univariable logistic regression, IMAT (odds ratio [OR] = 1.18, 95% CI [1.06–1.32]; *P* = 0.004) and SMFF (OR = 1.19, 95% CI [1.13–1.26]; *P* < 0.001) were associated with a higher risk of impaired glucose metabolism. This signal remained robust after multivariable adjustment for baseline demographics and cardiometabolic risk factors for SMFF (OR = 1.10, 95% CI [1.01–1.19]; *P* = 0.028) but not for IMAT (OR = 1.14, 95% CI [0.97–1.33]; *P* = 0.11).

**Conclusions:**

Quantitative SMFF, but not IMAT, is an independent predictor of impaired glucose metabolism, and discrimination is not significantly different from BMI, making it a promising alternative for the currently established approach. Automated methods such as the proposed model may provide a feasible option for opportunistic screening of myosteatosis and, thus, a low‐cost personalized risk assessment solution.

## Introduction

Type 2 diabetes (T2D) is a global health concern affecting 536.6 million individuals and is expected to further increase, posing a major socioeconomic challenge for public health and healthcare systems.^1^ As early states of impaired glucose metabolism can be reversed by lifestyle changes, physical activity and dietary habits, identifying individuals at risk is paramount to initiate such preventive measures to delay the onset of disease‐related complications and reduce morbidity and mortality.^2^

Currently, several invasive blood tests (e.g., A1C, fasting plasma glucose and oral glucose tolerance test [OGTT]) are available for the diagnosis of impaired glucose metabolism.^3^ However, they are not routinely performed in population‐based screening, and individuals in early subclinical stages of disease are often not aware of the increased risk, resulting in delayed diagnosis with potentially irreversible complications.^3^ In this context, medical imaging might be a promising tool for opportunistic risk assessment in daily routine based on alternations of anatomy that may become apparent before clinical symptoms occur.^4^ In particular, changes in body composition may play a crucial role as skeletal muscle (SM) fatty infiltration (i.e., myosteatosis) is associated with muscle deterioration and represents a unique ectopic adipose tissue depot.^5^ Both SM and adipose tissue compartments are important targets of insulin.^6^, ^7^, ^8^ For example, changes in intermuscular and intramuscular adipose tissue (IMAT) content, which are macroscopically visible on imaging studies such as computed tomography (CT) or magnetic resonance imaging (MRI), may be associated with impaired glucose metabolism and progression of insulin resistance.^7^ In addition, intramyocellular lipids have been shown to display metabolic activity by producing proinflammatory mediators, which may aggravate SM insulin resistance.^6^ However, unlike IMAT, which represents the macroscopically visible areas of adipose tissue between myofibrils, the intramyocellular adipose tissue compartment is not macroscopically quantifiable on traditional morphological MRI sequences. In contrast, chemical shift encoding‐based water–fat MRI, such as the Dixon technique that is widely used in routine clinical MRI studies, allows for quantitative measurements of the biochemical water–fat composition of SM, that is, SM fat fraction (SMFF), an estimate of myosteatosis, which may be more accurate than the currently established method of segmenting the amount of visible IMAT as it additionally quantifies the metabolically active intramyocellular adipose tissue.^9^, ^10^, ^11^, ^12^

However, quantification of these measures is not routinely performed because it would require an elaborate manual region of interest (ROI)‐based segmentation of IMAT and SM, which is time‐consuming and expensive as it disrupts the current established radiology workflow, even when performed on a single 2D slice.^13^ With recent advances in artificial intelligence, new methods for fully automated 3D estimation of imaging biomarkers such as SM and IMAT from routinely acquired imaging data have become available.^14^ Such automated imaging tasks may be translated into large‐scale applications in the opportunistic screening of routine scans to identify individuals at risk to guide clinical decision‐making.^15^

Here, we developed and tested a fully automated 3D deep learning model to estimate myosteatosis from whole‐body MRI using two different approaches: (1) macroscopically visible adipose tissue deposits and (2) quantitative SMFF. Subsequently, we investigated and compared the two measures and their discrimination for and association with impaired glucose metabolism as a potential tool for opportunistic population‐based risk assessment.

## Materials and methods

### Development of the deep learning model

**Figure 1:**
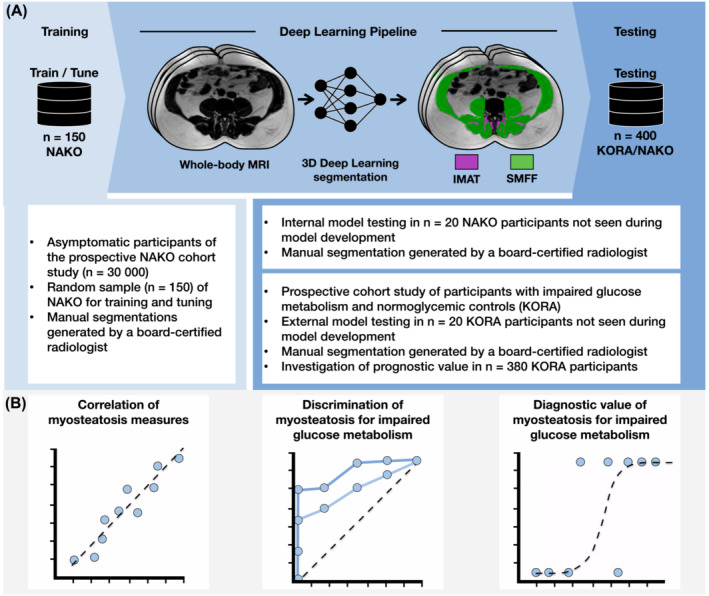
Overview of the study design. (A) A fully automated 3D deep learning model was developed and tested to estimate myosteatosis on T1‐weighted Dixon magnetic resonance imaging as (1) intramuscular adipose tissue (IMAT) and (2) quantitative skeletal muscle fat fraction (SMFF) using data from two prospective cohort studies. (B) Correlation, discrimination and prognostic value of the two myosteatosis measures were compared and investigated in individuals with impaired glucose metabolism and normoglycaemic controls. KORA, Cooperative Health Research in the Region of Augsburg; NAKO, German National Cohort.

An overview of the study design is provided in *Figure* *1*. We developed a fully automated deep learning model to volumetrically segment IMAT and SM on whole‐body T1‐weighted dual‐echo VIBE Dixon MRI. The model was developed using data from the German National Cohort (NAKO) study, an ongoing interdisciplinary, epidemiological cohort study that included 200 000 asymptomatic participants aged 20–72 years enrolled at 18 sites across Germany to investigate disease prevention and prognostication with a focus on major disease groups including cardiovascular disease, diabetes and cancer.^16^ In a sub‐study, 30 000 participants underwent additional whole‐body MRI as part of the study protocol (3T MAGNETOM Skyra, Siemens Healthineers, Erlangen, Germany). For the current project, a random sample of 150 participants from the imaging sub‐study was used for model training.

Manual annotations of IMAT and SM were performed on T1‐weighted Dixon sequences by an experienced radiologist blinded for clinical parameters (M.J., 5 years of experience in MRI) using all four imaging contrasts (water, fat, in‐phase and opposed‐phase) to facilitate the most accurate discrimination and segmentation of the different tissue boundaries. This is especially important in areas of fat–water interfaces, for example, between SM and fat tissue, where chemical shift artefacts of the second kind (also known as India ink or black line artefact) can occur in the opposed‐phase contrast, which can lead to overestimation or underestimation of tissue borders.^17^ In addition, all segmentations were independently validated and adapted, if necessary, by a board‐certified radiologist (J.W., 10 years of experience in MRI). IMAT segmentation was performed from the upper plate of the first thoracic vertebrae to the sacrum within the autochthonous spine musculature, which is a commonly used approach to quantify IMAT.^12^, ^18^ SM included all muscles of the trunk, pelvis and proximal thigh within the deep peripheral fascia from the upper plate of the first thoracic vertebrae to the femoral insertion of the adductor brevis muscle (*Figure* *S1*). All annotations were performed using the open‐source Nora medical imaging platform (www.nora‐imaging.org, Freiburg, Germany).

The only input to the proposed model was the in‐phase and opposed‐phase images of the whole‐body T1‐weighted dual‐echo VIBE Dixon MRI sequence; the output of the model was a segmentation mask estimating the volume (mL) of IMAT and SM on the entire scans as defined above. The model was implemented as a hierarchical, patch‐based stack of convolutional neural networks (CNNs). The patchwork approach^19^ is using nested patches of a fixed matrix size that decrease in physical size. A U‐Net‐type architecture is utilized in each scale, with the U‐Net matrix size set to 32 × 32 × 32 voxels for all scales, and a scale pyramid with a depth of four is employed. These settings were chosen based on the hardware capacity available, which limited the sample size and pyramid size. The pyramid size was chosen to achieve a coarsest layer field of view of 300 × 200 × 500 mm and a final isotropic resolution of 2 mm. Intermediate levels of the pyramid were exponentially interpolated. The input to the network consists of concatenated in‐phase and opposed‐phase contrasts. The architecture of the U‐Net used is similar to the default U‐Net configuration presented in the literature,^20^ with feature dimensions (32, 32, 64, 64, 128) and max pooling and transposed convolutions in the encoding and decoding layers, respectively. Each U‐Net has *n* + 8 output channels, with the first *n* = 4 corresponding to the labels and used for intermediate loss computations. The total logits of *n* + 8 outputs are passed to the next scale. The network is trained with the Adam optimizer^21^ with a rate of 0.001 and using 10 million patches for training of the network with a batch size of 32. The training took approximately 4 days. No systematic tuning was performed, and all labels were trained using binary cross‐entropy per channel. More details about the CNN architecture are reported elsewhere.^19^

### Independent testing of the deep learning model

The model was tested on two independent datasets not seen during any part of model development. All results reported in this study are for the testing datasets only.

The first testing dataset consisted of a random sample of 20 NAKO participants. The second testing dataset included 20 random participants from the ‘Cooperative Health Research in the Region of Augsburg’ (KORA) MRI sub‐study, an imaging study nested in the main prospective KORA FF4 cohort that enrolled 400 individuals (age 39–73 years) for whole‐body MRI assessment between June 2013 and September 2014 (3T MAGNETOM Skyra, Siemens Healthineers, Erlangen, Germany).^22^, ^23^ Inclusion criteria were as follows: consent to undergo whole‐body MRI and classification into prediabetes, diabetes or control. Exclusion criteria were as follows: age >73 years; subjects with stroke, myocardial infarction or revascularization; individuals with a cardiac pacemaker or implantable defibrillator, cerebral aneurysm clip, neural stimulator, any type of ear implant, an ocular foreign body or any implanted device; pregnant or breast‐feeding females; and subjects with claustrophobia, known allergy to gadolinium compounds or serum creatinine level ≥1.3 mg/dL.^23^

To evaluate model performance, the automatically generated 3D IMAT and SM segmentations of the model were compared to the manual segmentations using the Dice coefficient and Pearson's correlation coefficient. In addition, the quality of the automatic deep learning‐generated segmentations was visually assessed by an experienced radiologist.

### Assessment of myosteatosis

Myosteatosis, a surrogate for muscle quality, was estimated using two different approaches: (1) the traditional method by segmenting all macroscopically visible adipose tissue deposits within the autochthonous spinal musculature and (2) calculating the quantitative SMFF of all musculature depicted in the scan.

Currently, the first approach represents the established method, which is usually performed manually by segmenting macroscopically visible adipose tissue deposits in and between the autochthonous spinal musculature (IMAT) on a single slice at the height of the L3 vertebra using semi‐automatic software given the strong correlation between a single slice and whole‐body volumes.^24^ To mimic this approach but increase accuracy, we estimated IMAT within the autochthonous spinal musculature on the entire whole‐body scan. To capture and normalize for differences in individual muscle mass, a summary measure of IMAT was calculated for further analyses using the following equation:

$$\text{IMAT}=\frac{\text{IMAT volume}\;\left(\mathrm{mL}\right)}{\mathrm{SM}\;\text{volume}\;\left(\mathrm{mL}\right)}\cdot 100$$

In contrast to this relative and crude estimation of myosteatosis, chemical shift encoding‐based water–fat MRI allows for a quantitative assessment of myosteatosis exploiting the acquired water–fat information to estimate the SMFF. Based on the whole‐body 3D SM segmentation masks, water and fat selective images of the dual‐echo Dixon VIBE sequence (slice thickness of 1.7 mm, voxel size of 1.7 × 1.7 mm, field of view of 488 × 716 mm, matrix of 256 × 256, repetition time [TR] of 4.06 ms, echo time [TE] of 1.26 and 2.49 ms, and flip angle of 9°) were used to calculate SMFF as follows:

$$\text{SMFF}=\frac{\text{mean intensity}\;\mathrm{fat}\;\text{image}}{\text{mean intensity}\;\mathrm{fat}\;\text{image}+\text{mean intensity water image}}$$

### Discrimination and diagnostic performance of myosteatosis

**Figure 2:**
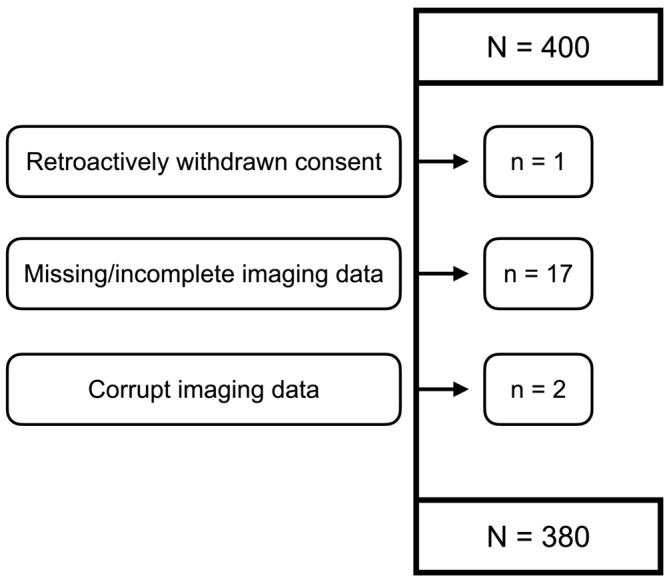
Participant flowchart.

In this retrospective study, we applied the 3D deep learning model to estimate IMAT and SMFF in all participants enrolled in the KORA MRI sub‐study to investigate and compare the two myosteatosis measures regarding their discrimination for and association with impaired glucose metabolism. Two individuals had to be excluded due to poor image quality and/or corrupted imaging data. Additional 17 participants were excluded because of missing or incomplete imaging data, and one withdrawn consent to the study, resulting in a final study cohort of 380 individuals (*Figure* *2*). Retrospective secondary use of the KORA MRI sub‐study data was approved by the local institutional review board (Ethics Committee, University of Freiburg, EK‐Nr.: 22‐1336‐S1‐retro).

### Clinical covariates

All participants underwent a comprehensive health assessment at the study centre with standardized interviews as well as physical and laboratory examinations for demographic and cardiometabolic risk assessment. Cardiometabolic risk factors comprised the following: age, sex, body mass index (BMI) (kg/m^2^), cholesterol levels (mg/dL), high‐density lipoprotein (HDL) levels (mg/dL), low‐density lipoprotein (LDL) levels (mg/dL), triglyceride levels (mg/dL), alcohol consumption (g/day), systolic blood pressure (mmHg) and smoking status (current, former and never).

### Outcome

The primary outcome of this study was impaired glucose metabolism, which was defined as prevalent T2D or determined based on a 75‐g OGTT after at least 8 h of overnight fasting and defined as impaired fasting glucose (IFG: fasting glucose 110–125 mg/dL), impaired glucose tolerance (IGT: 2‐h glucose 140–200 mg/dL) or newly diagnosed diabetes mellitus (≥125‐mg/dL fasting and/or ≥200‐mg/dL 2‐h glucose load) according to World Health Organization (WHO) criteria.^25^

### Statistical analysis

Baseline characteristics of the study sample are presented as mean ± standard deviation (SD) or median with inter‐quartile ranges (IQRs) for continuous variables and absolute counts with percentages for categorical variables. Differences between individuals with impaired glucose metabolism and normoglycaemic controls were assessed using the *t* test, Mann–Whitney *U* test or *χ* ^2^ test, as appropriate.

Correlations between IMAT and SMFF were evaluated using Pearson's correlation coefficient. The discriminatory capacity of IMAT, SMFF and traditional risk factors was assessed by calculating areas under the receiver operating characteristic curve (AUCs) and compared using DeLong's test.^26^ The association between IMAT and SMFF with impaired glucose metabolism was assessed via univariable and multivariable logistic regression analysis adjusted for baseline demographics and cardiometabolic risk factors (lipid panel, systolic blood pressure, smoking status and alcohol consumption) as specified above. Statistical significance was indicated by *P* values <0.05. Statistical analysis was performed using R Version 4.2.1 (R Core Team, www.r‐project.org, 2022).

## Results

### Independent internal testing of the deep learning model

To evaluate the performance of the proposed deep learning system for volumetric quantification of IMAT and SM, we tested the model on two independent test datasets not seen during model development by comparing the automatic and manual volume measurements.

Internal testing was performed on 20 individuals of the NAKO, where we found a Dice coefficient of 0.83 ± 0.7 for IMAT and 0.91 ± 0.03 for SM and Pearson's correlation coefficients of 0.98 and 0.99 (all *P* values <0.001), respectively.

**Figure 3:**
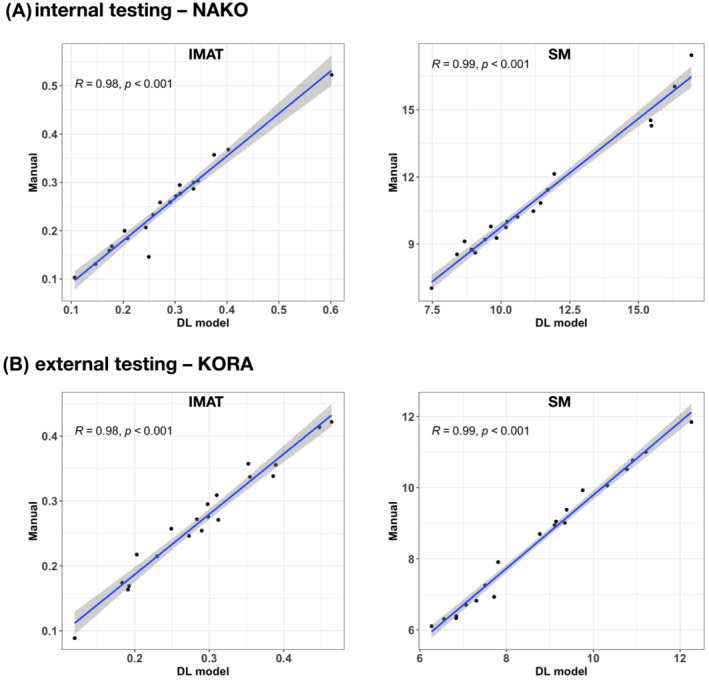
Correlation between manual and deep learning (DL)‐quantified intramuscular adipose tissue (IMAT) and skeletal muscle (SM) volumes in the independent testing datasets. We observed a high correlation between the 3D segmentation mask volumes of IMAT and SM in (A) the internal and (B) external testing datasets. KORA, Cooperative Health Research in the Region of Augsburg; NAKO, German National Cohort.

External testing was performed on 20 participants of the KORA imaging sub‐study without any retraining. The Dice coefficient was 0.81 ± 0.03 for IMAT and 0.93 ± 0.03 for SM, and Pearson's correlation coefficient was 0.98 and 0.99 (all *P* values <0.001), respectively (*Figure* *3*).

In addition, no systematic errors with respect to age, sex or BMI were noted across all study participants in the visual assessment.

### Diagnostic performance of myosteatosis

The final study sample comprised 380 participants (162 women and 218 men) with a mean age of 56.2 ± 9.2 years and a mean BMI of 28.2 ± 4.9 kg/m^2^ (*Table* *1*). Individuals with impaired glucose metabolism (*n* = 146 [38.4%]) were older (59.2 ± 8.7 vs. 54.3 ± 8.9 years) and more likely men (68% vs. 50%) and had a higher BMI (30.6 ± 5.1 vs. 26.7 ± 4.2 kg/m^2^), lower HDL levels (55.9 ± 15.4 vs. 65.6 ± 18.0 mg/dL), higher triglycerides (173.3 ± 100.1 vs. 107.1 ± 64.0 mg/dL) and higher systolic blood pressure (127.6 ± 17.6 vs. 116.5 ± 15.0 mmHg; all *P* < 0.001; *Table* *1*) compared to individuals with normoglycaemia.

**Table 1:** Baseline demographics and cardiometabolic risk factors

	Entire cohort	Normoglycaemia	Impaired glucose metabolism	*P* value^b^
Individuals	*N* = 380 ^a^	*N* = 234 ^a^	*N* = 146 ^a^	
Age (years)	56.2 ± 9.2	54.3 ± 8.9	59.2 ± 8.7	**<0.001**
Female	162 (43%)	116 (50%)	46 (32%)	**<0.001**
Weight (kg)	83.2 ± 16.6	78.5 ± 15.3	90.7 ± 15.8	**<0.001**
BMI (kg/m ^2^ )	28.2 ± 4.9	26.7 ± 4.2	30.6 ± 5.1	**<0.001**
IMAT (%)	4.1 ± 1.9	3.9 ± 1.7	4.5 ± 2.2	**0.005**
SMFF (%)	20.1 ± 4.4	18.9 ± 3.9	22.0 ± 4.7	**<0.001**
Cholesterol (mg/dL)	218.4 ± 36.6	216.5 ± 35.9	221.3 ± 37.5	0.2
HDL (mg/dL)	61.9 ± 17.7	65.6 ± 18.0	55.9 ± 15.4	**<0.001**
LDL (mg/dL)	140.0 ± 33.1	138.7 ± 32.1	142.1 ± 34.7	0.3
Triglyceride (mg/dL)	132.5 ± 86.0	107.1 ± 64.0	173.3 ± 100.1	**<0.001**
Alcohol consumption (g/day)	18.5 ± 24.1	16.7 ± 21.7	21.5 ± 27.3	0.068
Systolic blood pressure (mmHg)	120.8 ± 16.9	116.5 ± 15.0	127.6 ± 17.6	**<0.001**
Smoking				0.12
Current smokers	76 (20%)	52 (22%)	24 (17%)	
Former smokers	166 (44%)	91 (39%)	75 (51%)	
Never smokers	138 (36%)	91 (39%)	47 (32%)	

*Note*: Bold indicates a statistically significant difference between normoglycemic controls and individuals with impaired glucose metabolism.

Abbreviations: BMI, body mass index; HDL, high‐density lipoprotein; IMAT, intramuscular adipose tissue; LDL, low‐density lipoprotein; SMFF, skeletal muscle fat fraction.

^a^ Mean ± SD and *n* (%).

^b^ Welch two‐sample *t* test and Pearson's *χ* ^2^ test.

**Figure 4:**
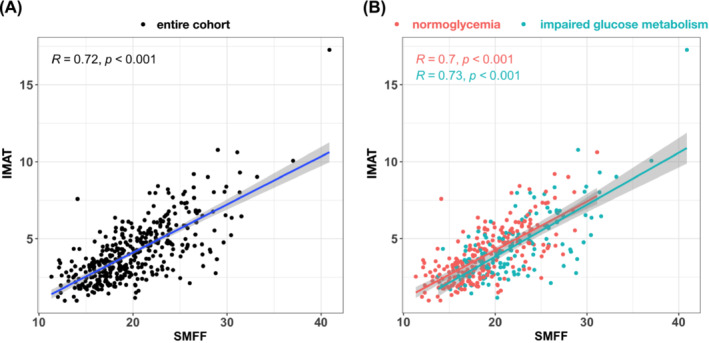
Correlation between intramuscular adipose tissue (IMAT) and skeletal muscle fat fraction (SMFF) (A) in the entire cohort and (B) stratified by impaired glucose metabolism.

IMAT was significantly higher in individuals with impaired glucose metabolism (4.5 ± 2.2%) compared to those without impaired glucose metabolism (3.9 ± 1.7%; *P* = 0.005). A similar pattern was observed for SMFF (22.0 ± 4.7% vs. 18.9 ± 3.9%; *P* < 0.001; *Table* *1* and *Figure* *S2*). The correlation between IMAT and SMFF was high (*r* = 0.72, *P* < 0.001; *Figure* *4* *A*) and did not differ after stratification for the presence of impaired glucose metabolism (*r* = 0.7 and 0.73; *P* < 0.001, *Figure* *4* *B*).

#### Discriminative performance of myosteatosis

**Figure 5:**
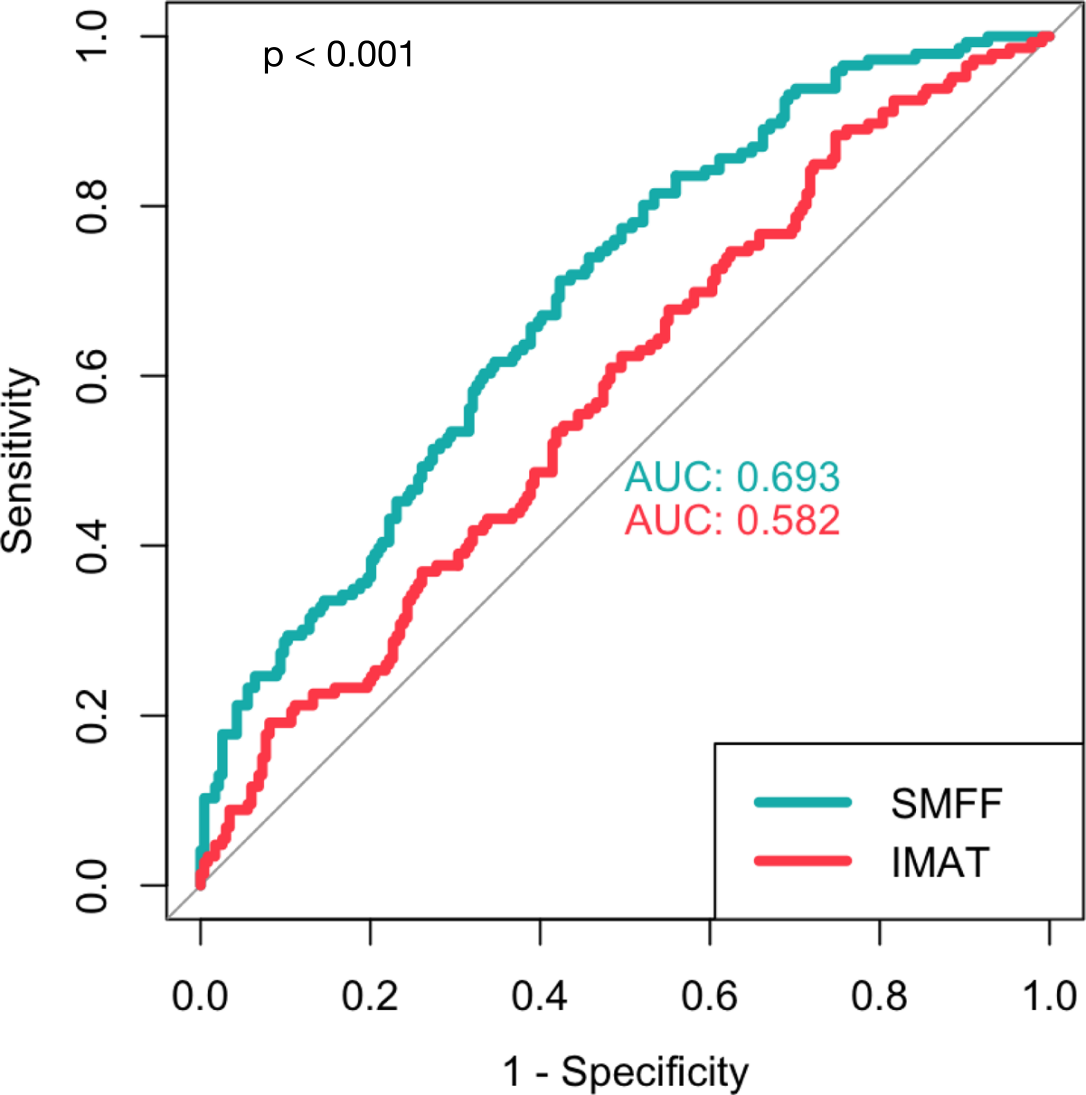
Discrimination of the two myosteatosis measures (intramuscular adipose tissue [IMAT] and skeletal muscle fat fraction [SMFF]) for impaired glucose metabolism. Quantitative SMFF had a significantly higher discriminatory capacity for impaired glucose metabolism than IMAT (area under the receiver operating characteristic curve [AUC] 0.693 vs. 0.582, 95% CI [0.06–0.16]; *P* < 0.001).

Quantitative SMFF had higher discrimination for impaired glucose metabolism than the IMAT (AUC 0.693 vs. 0.582, 95% confidence interval [CI] [0.06–0.16]; *P* < 0.001; *Figure* *5*). Considering traditional demographic (age, sex and BMI) and cardiometabolic risk factors (lipid panel, systolic blood pressure, smoking status and alcohol consumption), we observed slightly but not statistically significant higher AUCs for BMI (AUC 0.733 vs. 0.693, 95% CI [−0.09 to 0.01]; *P* = 0.15) and triglycerides (0.756 vs. 0.693, 95% CI [−0.13 to 0.01]; *P* = 0.08; *Figure* *S3*).

#### Association between myosteatosis and impaired glucose metabolism

**Figure 6:**
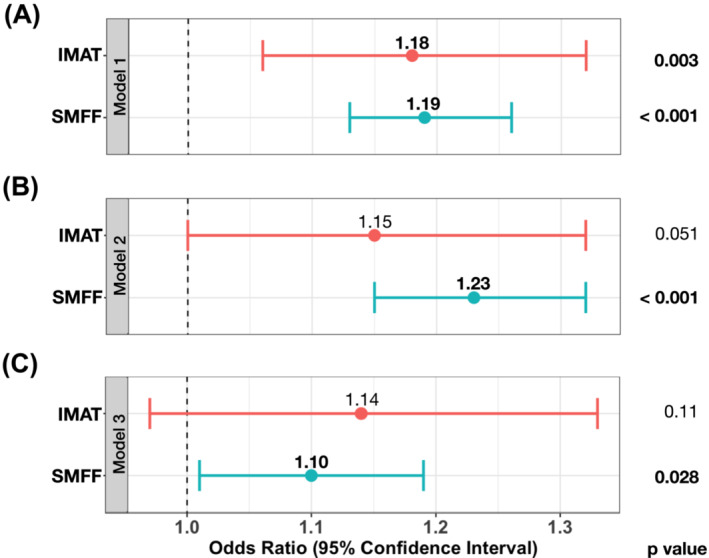
Association between intramuscular adipose tissue (IMAT) and skeletal muscle fat fraction (SMFF) and impaired glucose metabolism. (A) Univariable models and multivariable‐adjusted models for (B) age and sex and (C) baseline demographics (age, sex and body mass index) and cardiometabolic risk factors (lipid panel, systolic blood pressure, smoking status and alcohol consumption) with odds ratios and 95% confidence intervals. After multivariable adjustment, only SMFF remained as an independent predictor of impaired glucose metabolism, whereas the signal for skeletal muscle was attenuated.

In univariable logistic regression analysis, IMAT and SMFF were associated with a higher risk of impaired glucose metabolism (odds ratio [OR] = 1.18, 95% CI [1.06–1.32]; *P* = 0.004; and OR = 1.19, 95% CI [1.13–1.26]; *P* < 0.001, respectively; *Figure* *6* *A*). This signal remained robust for SMFF in multivariable logistic regression analysis after adjusting for age and sex (OR = 1.23, 95% CI [1.15–1.32]; *P* < 0.001; *Figure* *6* *B*), whereas the signal for IMAT was attenuated (OR = 1.15, 95% CI [1.00–1.32]; *P* = 0.051; *Figure* *6* *B*). After further adjustment for baseline demographics (age, sex and BMI) and cardiometabolic risk factors (lipid panel, systolic blood pressure, smoking status and alcohol consumption), the association between SMFF and impaired glucose metabolism remained significant (OR = 1.10, 95% CI [1.01–1.19]; *P* = 0.028; *Figure* *6* *C*) but not for IMAT (OR = 1.14, 95% CI [0.97–1.33]; *P* = 0.11; *Figure* *6* *C*). The results of the logistic regression models are shown in *Tables* *S1* and *S2*.

## Discussion

In this study, we proposed a fully automated 3D deep learning framework that allows for robust and accurate quantification of myosteatosis from routine whole‐body MRI using two different approaches: macroscopically visible IMAT deposits and quantitative SMFF. We found that both measures of myosteatosis were associated with a higher risk of impaired glucose metabolism in a community‐dwelling population, but only the quantitative SMFF remained as an independent predictor after multivariable adjustment for cardiometabolic risk factors.

These results are of clinical importance as SM is the largest target organ of insulin and accounts for the majority of glucose uptake.^27^ In addition, it plays a key role in maintaining glucose homeostasis and insulin resistance.^28^ In particular, fatty infiltration of SM—that is, myosteatosis—is considered an indicator of muscle quality with direct implications on the glucose metabolism mediating capabilities and the development of T2D.^29^, ^30^ Consequently, several medical imaging‐based approaches have been proposed to estimate myosteatosis from SM segmentation masks, including Hounsfield unit (HU)‐based assessment on CT and quantitative imaging techniques on MRI.^31^ While the first relies on relatively coarse density (HU) changes of the muscle due to accumulating adipose tissue, MRI allows for a more accurate voxel‐wise quantitative assessment using chemical shift encoding‐based water–fat MRI.^9^, ^32^ Traditionally, myosteatosis is estimated manually on a single slice to reduce labour‐intensive segmentation given the high correlation with whole‐body volumes.^24^ Yet this approach is known to reduce accuracy compared with whole‐body volumetric measurements.^33^ With advances in artificial intelligence, automated and more accurate 3D segmentation approaches have become feasible^14^ with the potential to elucidate associations that were unknown thus far.

For the interpretation of the results presented in this study, it is important to keep in mind that the degree of myosteatosis depends on two components: (1) intermuscular adipose tissue and IMAT, which can be depicted as macroscopically visible adipose tissue deposits between muscle groups and fibre,^7^ and (2) intramyocellular fat, which is not visible but can be detected by decreased attenuation on CT^34^ or increased SMFF on chemical shift encoding‐based water–fat MRI.^35^ Intramyocellular fat is well known to be related to metabolic dysfunction including impaired glucose metabolism and insulin homeostasis.^36^ In contrast, IMAT is considered to be rather metabolically inactive, yet several studies reported positive correlations between IMAT and insulin resistance.^7^, ^29^, ^30^ Although these are two substantially different concepts to estimate myosteatosis, they are both considered valid measures to describe fatty infiltration and muscle quality. The proposed model in this study allows for estimating both measures of myosteatosis: the traditional approach based on macroscopically visible intermuscular adipose tissue and IMAT deposits and the quantitative intramyocellular SMFF. Based on our results, we consider SMFF as the favourable measure, as segmentation of SM is possible with higher accuracy compared to the traditional IMAT approach. In addition, SMFF discriminated significantly better than IMAT for impaired glucose metabolism, although it was similar in discrimination to some of the traditional risk factors such as BMI and triglycerides. Further, in multivariable logistic regression analysis, only SMFF but not IMAT turned out to be independently associated with impaired glucose metabolism beyond traditional demographic and cardiometabolic risk factors. This highlights the importance of capturing metabolically active intramyocellular fat rather than relying on the traditional approach of macroscopically visible IMAT. These results are in line with previous studies and further support the potential of imaging‐based estimation of myosteatosis for opportunistic risk assessment of impaired glucose metabolism.^37^ As no human input is required, the proposed end‐to‐end pipeline could be implemented into clinical systems (e.g., picture archiving and communication system [PACS] or electronic medical record [EMR]) without disruption of daily workflows to automatically quantify readily available and prognostically relevant information on existing and new MRI examinations that may otherwise go unnoticed.

This study has limitations. First, the sample size of the datasets for model development and testing as well as clinical application was relatively small and limited to a central–western population. The generalizability of our results to more heterogeneous populations requires confirmation in larger prospective cohort studies. In addition, MRI‐based results of myosteatosis measurements were not compared to histopathology, which is still considered the gold standard for quantification of fat content. However, previous studies showed that a standardized quantification of SM fat by measurement of SMFF is a valid and reproducible approach to assess myosteatosis with a high correlation to histology.^12^, ^38^ Last, the dual‐echo Dixon MRI sequence used here for SMFF measurements did not account for T2* effects, therefore likely overestimating fat fraction when compared to other methods like 6‐point Dixon MRI or MR spectroscopy.^39^ Despite this, our analysis focused on relative differences between groups and was not focused on providing reference values. In addition, the use of a dual‐echo Dixon sequence facilitates clinical application, as this technique is more widely used due to shorter acquisition times compared to multi‐echo Dixon sequences in routine clinical examinations.^40^

In conclusion, deep learning allows for fully automated and reliable estimation of myosteatosis on whole‐body MRI. Quantitative chemical shift encoding‐based SMFF is an independent predictor of impaired glucose metabolism beyond traditional cardiometabolic risk factors, and discriminatory capacity is not significantly different from BMI. Thus, SMFF is a promising alternative to estimate myosteatosis compared to the current approach of macroscopically visible IMAT. The proposed pipeline may be helpful for opportunistic screening of impaired glucose metabolism to identify individuals at high risk and guide preventive lifestyle changes to improve population health.

## Conflict of interest statement

The authors declare no conflicts of interest.

## Supporting information


**Figure S1.** Volume rendering of the automated 3D deep learning model SM segmentation mask output. 3D volume rendering of the deep learning model SM segmentation mask output of a 44 year old male with a BMI of 29 kg/m^2^. SM included all muscles of the trunk, pelvis and proximal thigh within boundaries of the deep peripheral fascia from the upper plate of the first thoracic vertebrae to the femoral insertion of the adductor brevis muscle. BMI, body mass index. M, male. SM, skeletal muscle. T1, thoracic vertebrae 1.

**Figure S2.** 2D illustration of the 3D deep learning model segmentation mask outputs for IMAT, SM, and SM‐derived SMFF. IMAT and SMFF were significantly higher in individuals with impaired glucose metabolism compared to normoglycemic controls. (A) 60 year old normoglycemic male with a BMI of 29 kg/m^2^, IMAT of 3,3% and SMFF of 19.2%. (B) 69 year old male with a BMI of 30 kg/m^2^, IMAT of 6.7%, and SMFF of 26.7%. Upper row shows a single‐slice at lumbar vertebrae 3 of the Dixon fat contrast image (derived from the in‐phase and opposed‐phase contrasts that were used as model input). Middle row shows a 2D illustration of the 3D deep learning model segmentation mask outputs for IMAT (magenta) and SM (green) at the same height superimposed on the Dixon fat image. Lower row shows a 2D voxel‐wise illustration of SMFF, which was derived from the 3D SM segmentation mask superimposed on the Dixon fat image. Cold color indicates low SMFF, and hot color indicates high SMFF. BMI, body mass index. IMAT, intramuscular adipose tissue. M, male. SM, skeletal muscle. SMFF, skeletal muscle fat fraction.

**Figure S3.** Discrimination of baseline demographic and cardiometabolic risk factors for impaired glucose metabolism. Receiver operating characteristic curves and AUCs showing discrimination of the two myosteatosis measures (A) SMFF and (B) IMAT, baseline demographics (C) Sex, (D) Age, and (E) BMI, and cardiometabolic risk factors (F) total cholesterol, (G) HDL, (H) LDL, (I) triglycerides, (J) alcohol consumption, (K) systolic blood pressure, and (L) smoking status for impaired glucose metabolism. AUC, area under the curve. IMAT, intramuscular adipose tissue. SMFF, skeletal muscle fat fraction.

**Table S1.** Association between IMAT and impaired glucose metabolism.
**Table S2.** Association between SMFF and impaired glucose metabolism.
