# Supplementary figures and images for: Association between myosteatosis and impaired glucose metabolism: A deep learning whole‐body magnetic resonance imaging population phenotyping approach
Source: J Cachexia Sarcopenia Muscle. 2024 Jul 15;15(5):1750–60. doi: 10.1002/jcsm.13527 (PMC11446675; doi:10.1002/jcsm.13527)

upper plate T1

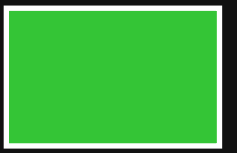

SM

Age  
44y

Sex  
M

BMI  
29 kg/m<sup>2</sup>

adductor brevis insertion

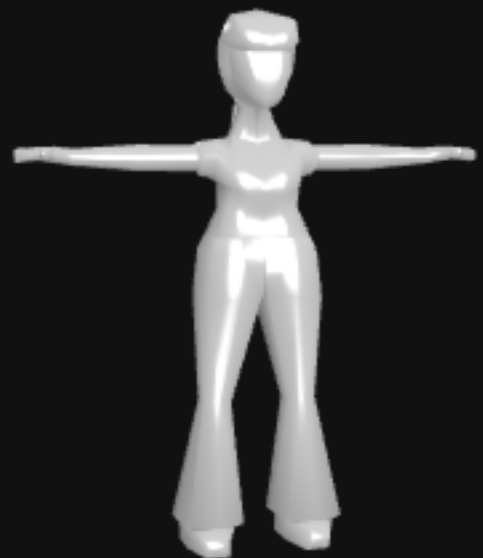

Supplement: Supplementary file 1 — Figure S1. Volume rendering of the automated 3D deep learning model SM segmentation mask output. 3D volume rendering of the deep learning model SM segmentation mask output of a 44 year old male with a BMI of 29 kg/m2. SM included all muscles of the trunk, pelvis and proximal thigh within boundaries of the deep peripheral fascia from the upper plate of the first thoracic vertebrae to the femoral insertion of the adductor brevis muscle. BMI, body mass index. M, male. SM, skeletal muscle. T1, thoracic vertebrae 1. [file JCSM-15-1750-s002.pdf]

Dixon  
Fat image

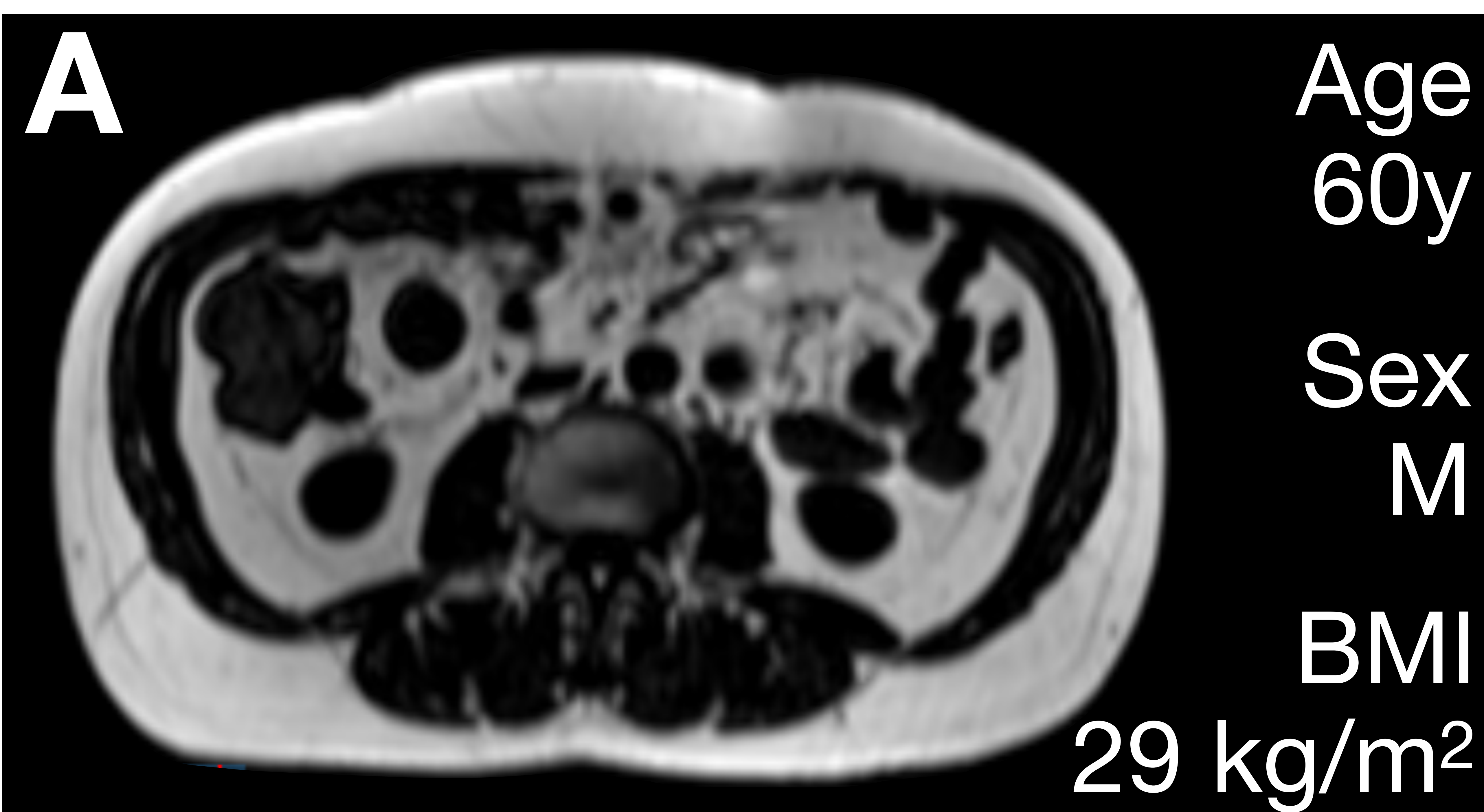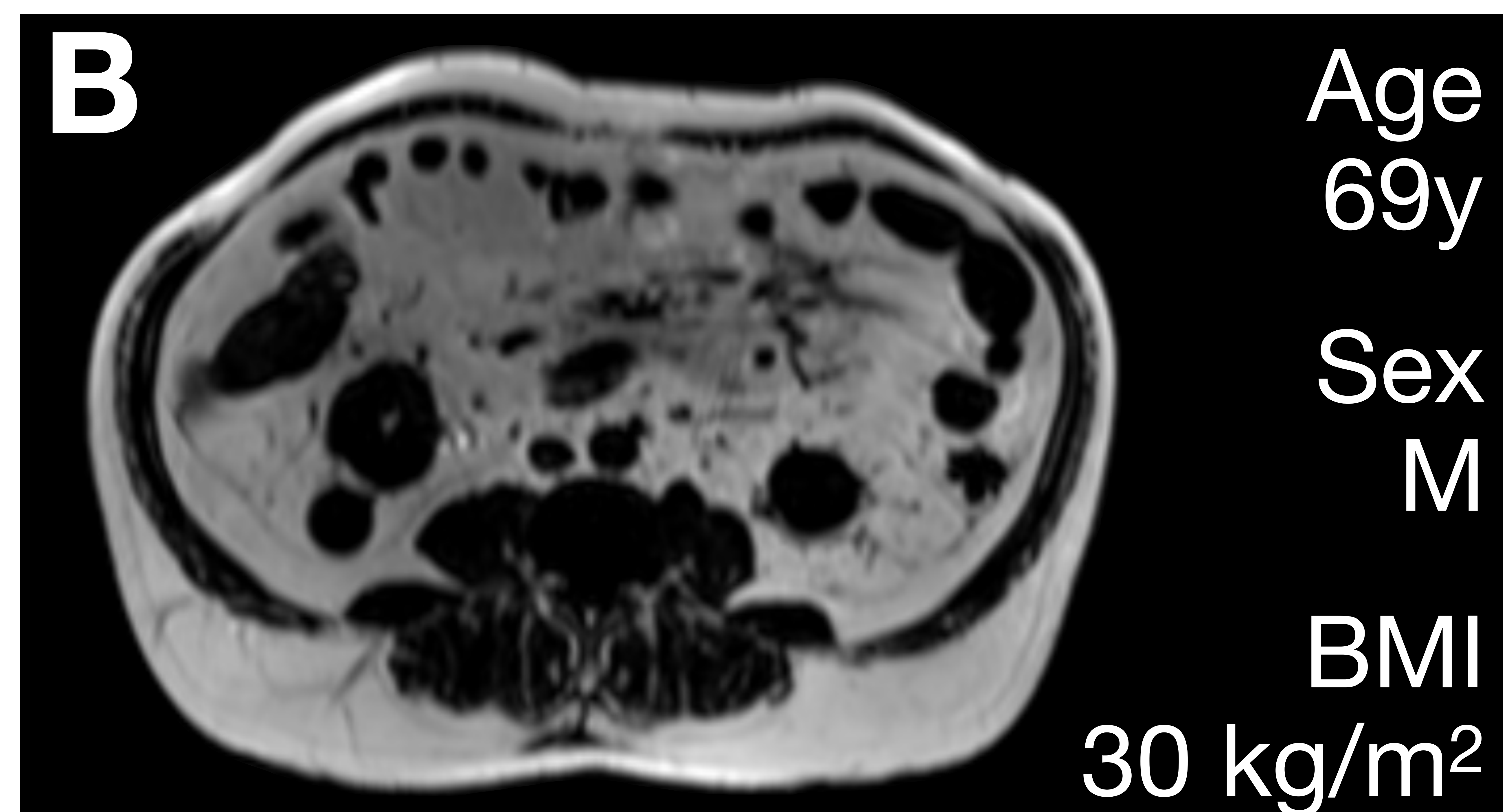

Output  
Deep learning  
segmentation  
masks

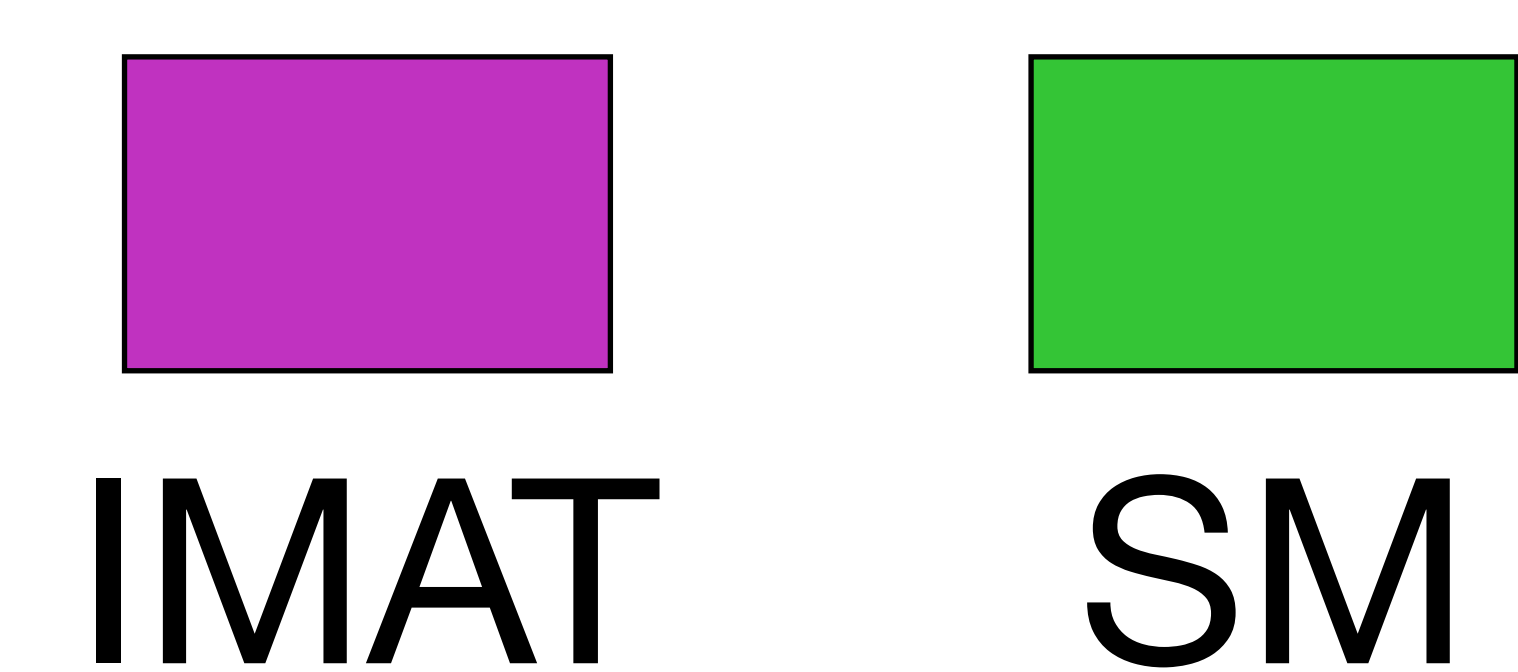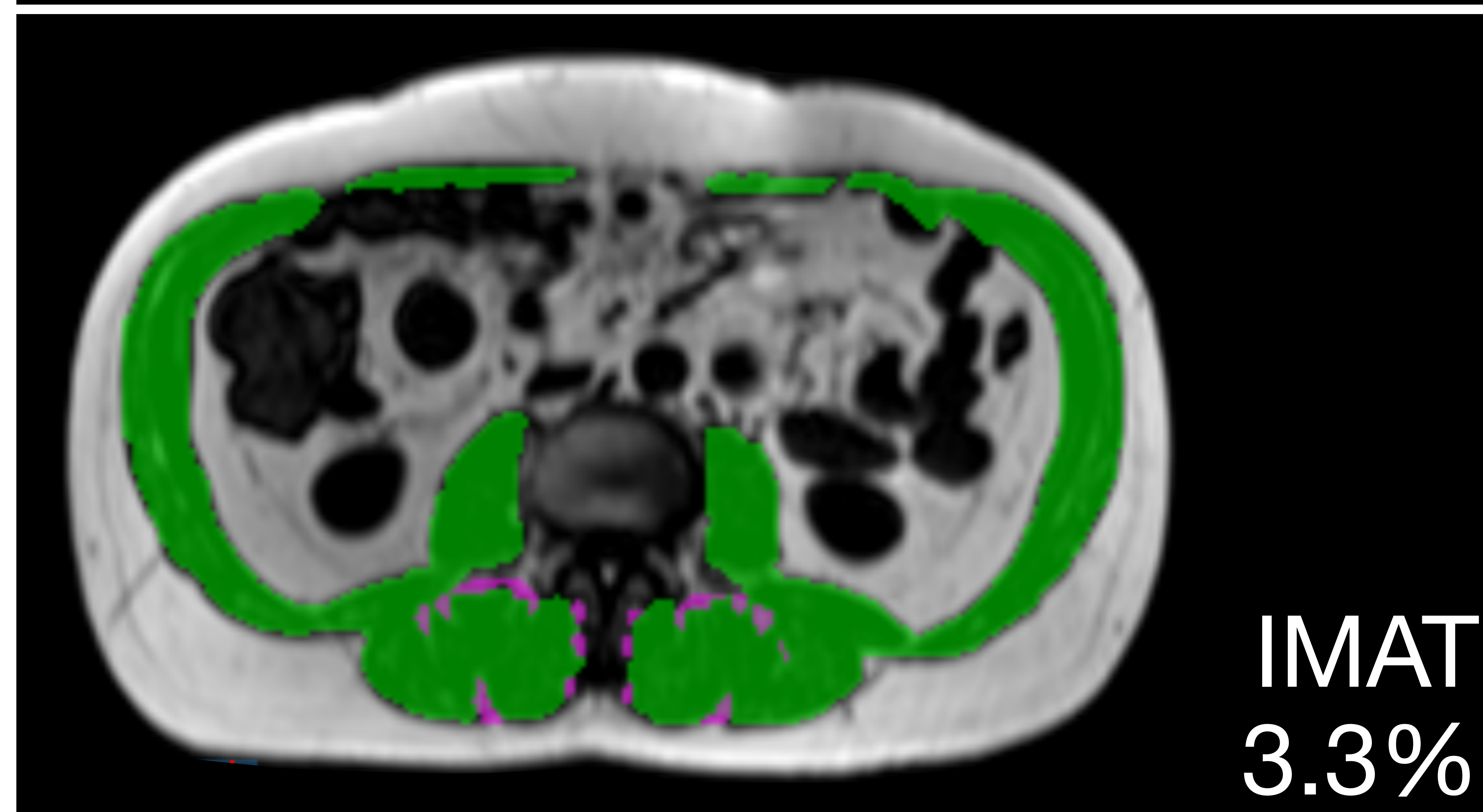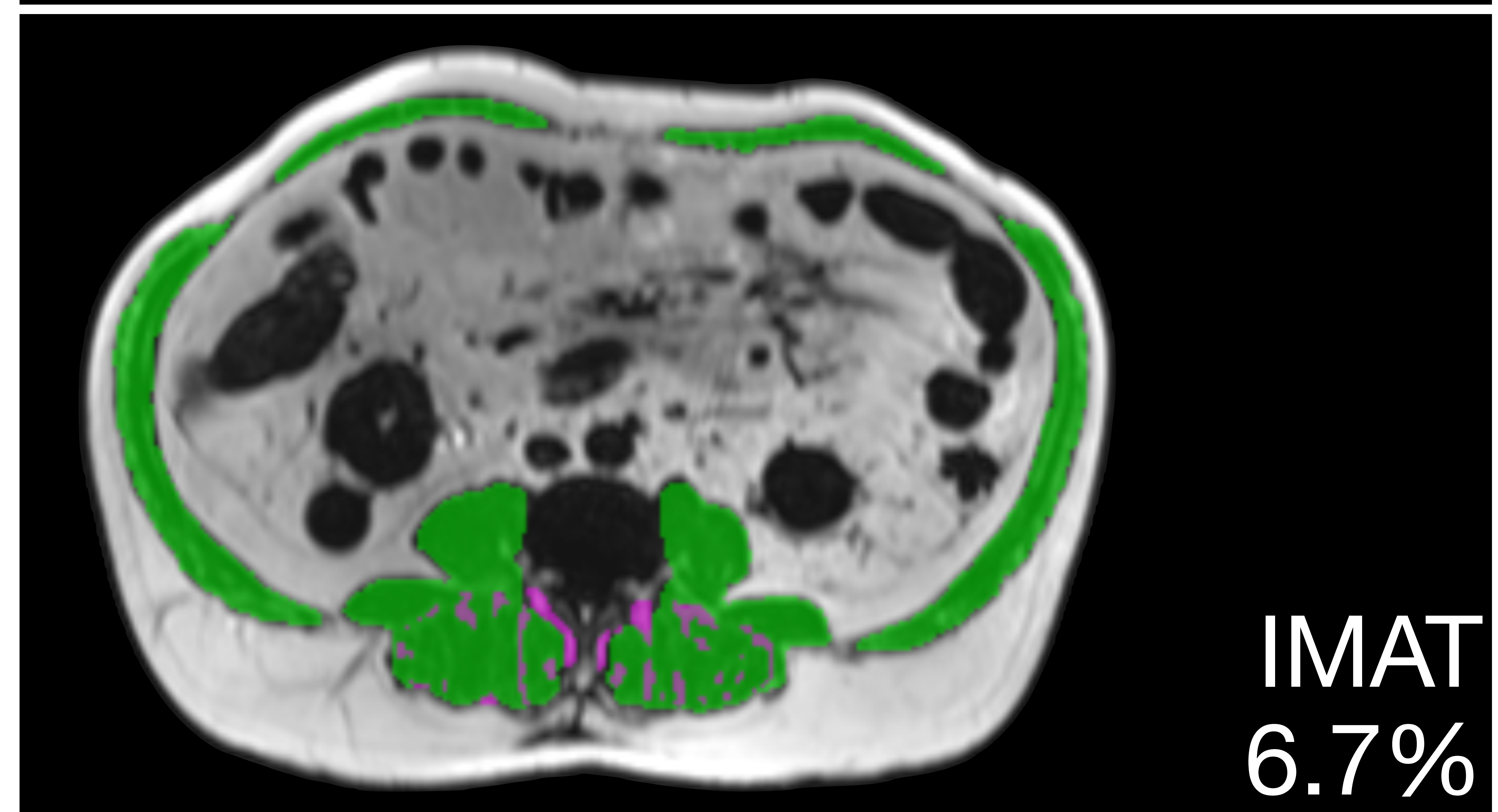

SM derived  
Dixon based  
SMFF %

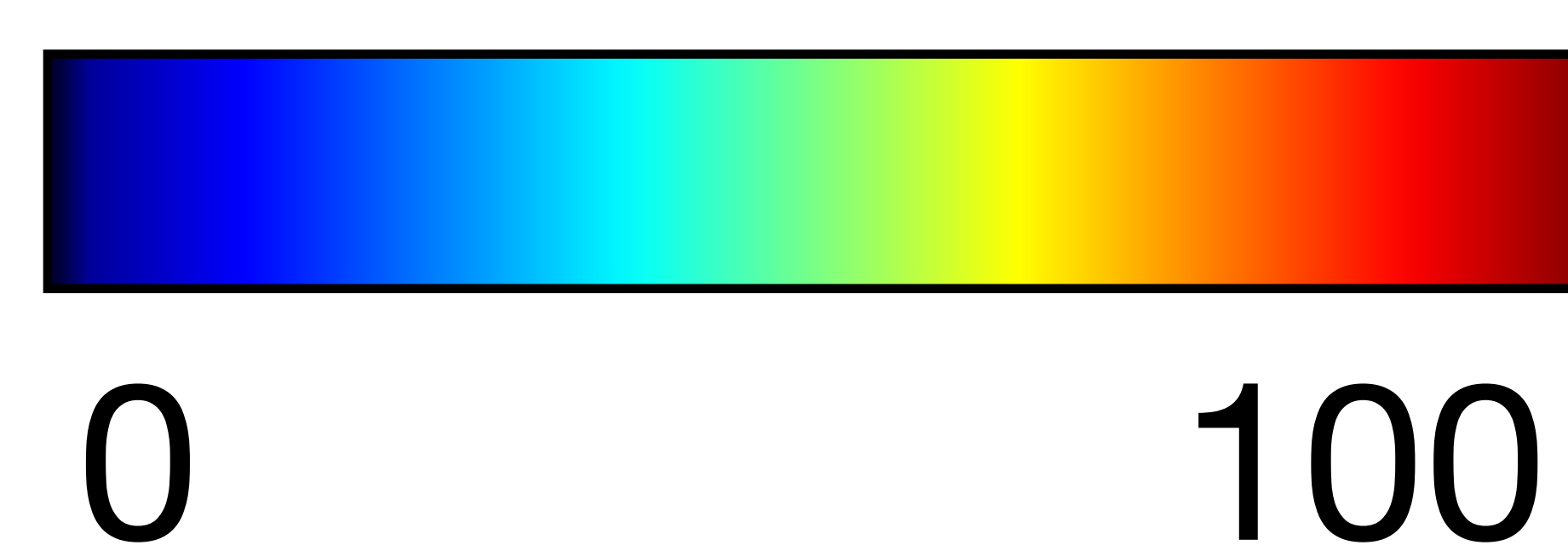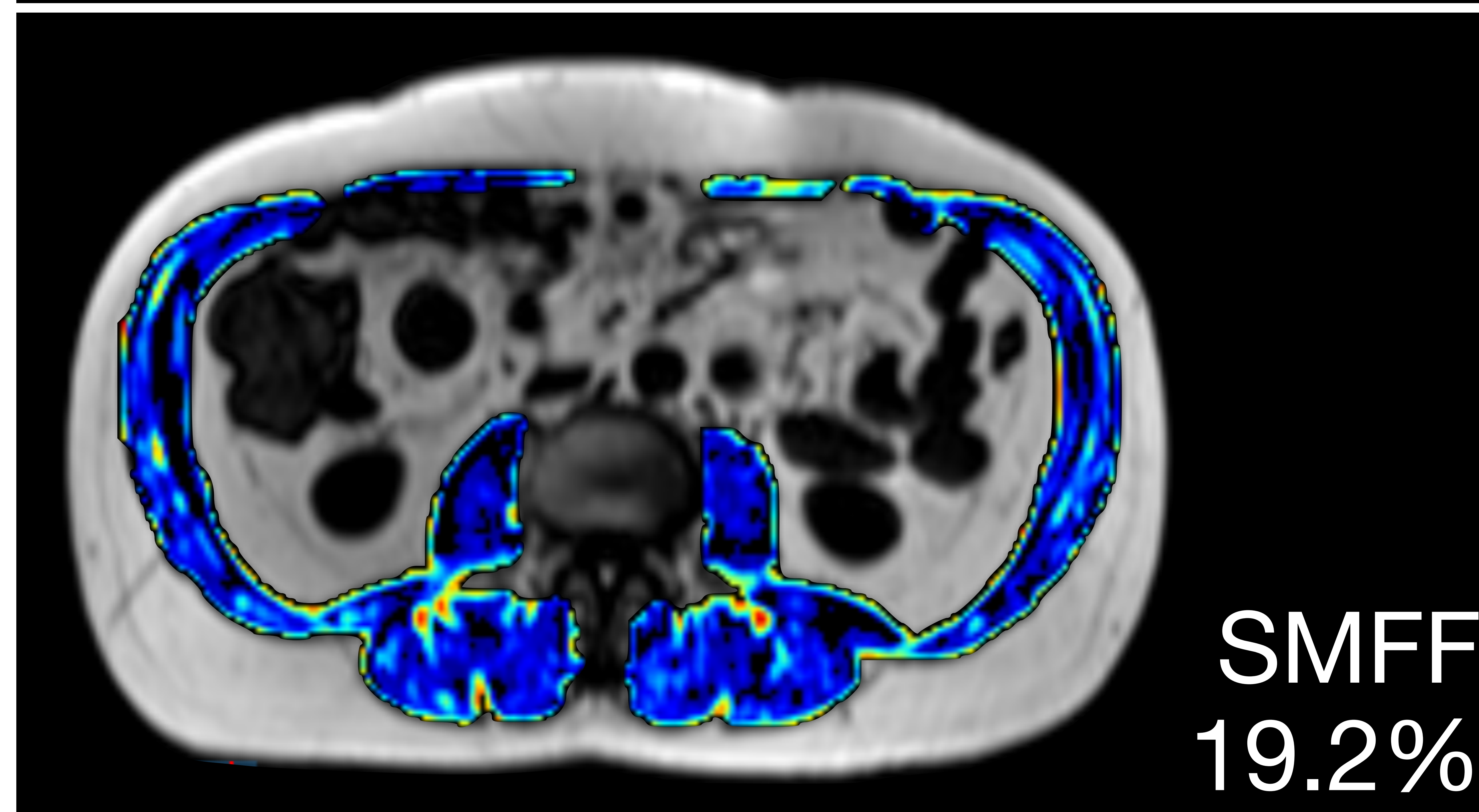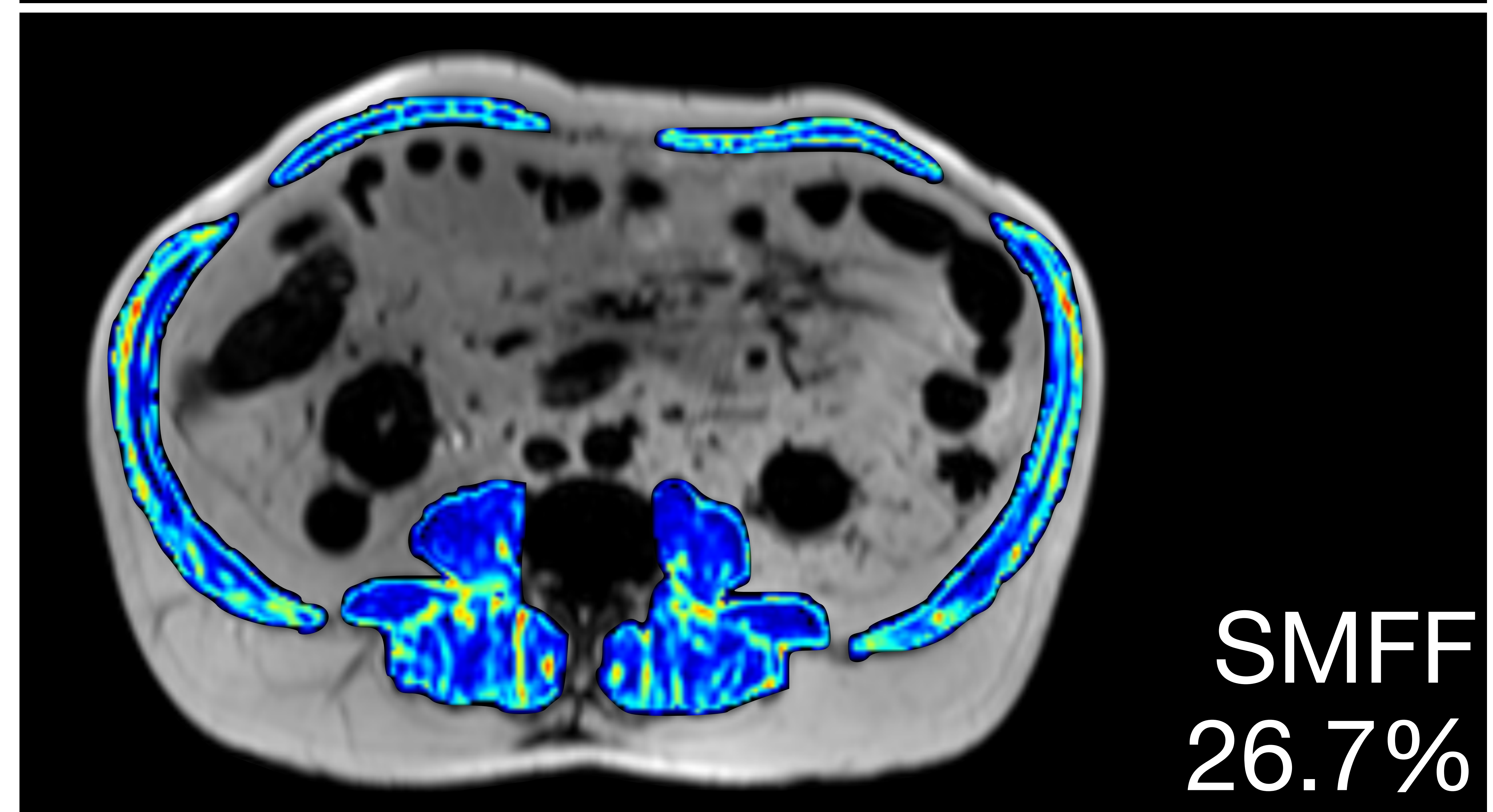

Supplement: Supplementary file 2 — Figure S2. 2D illustration of the 3D deep learning model segmentation mask outputs for IMAT, SM, and SM‐derived SMFF. IMAT and SMFF were significantly higher in individuals with impaired glucose metabolism compared to normoglycemic controls. (A) 60 year old normoglycemic male with a BMI of 29 kg/m2, IMAT of 3,3% and SMFF of 19.2%. (B) 69 year old male with a BMI of 30 kg/m2, IMAT of 6.7%, and SMFF of 26.7%. Upper row shows a single‐slice at lumbar vertebrae 3 of the Dixon fat contrast image (derived from the in‐phase and opposed‐phase contrasts that were used as model input). Middle row shows a 2D illustration of the 3D deep learning model segmentation mask outputs for IMAT (magenta) and SM (green) at the same height superimposed on the Dixon fat image. Lower row shows a 2D voxel‐wise illustration of SMFF, which was derived from the 3D SM segmentation mask superimposed on the Dixon fat image. Cold color indicates low SMFF, and hot color indicates high SMFF. BMI, body mass index. IMAT, intramuscular adipose tissue. M, male. SM, skeletal muscle. SMFF, skeletal muscle fat fraction. [file JCSM-15-1750-s003.pdf]

**A**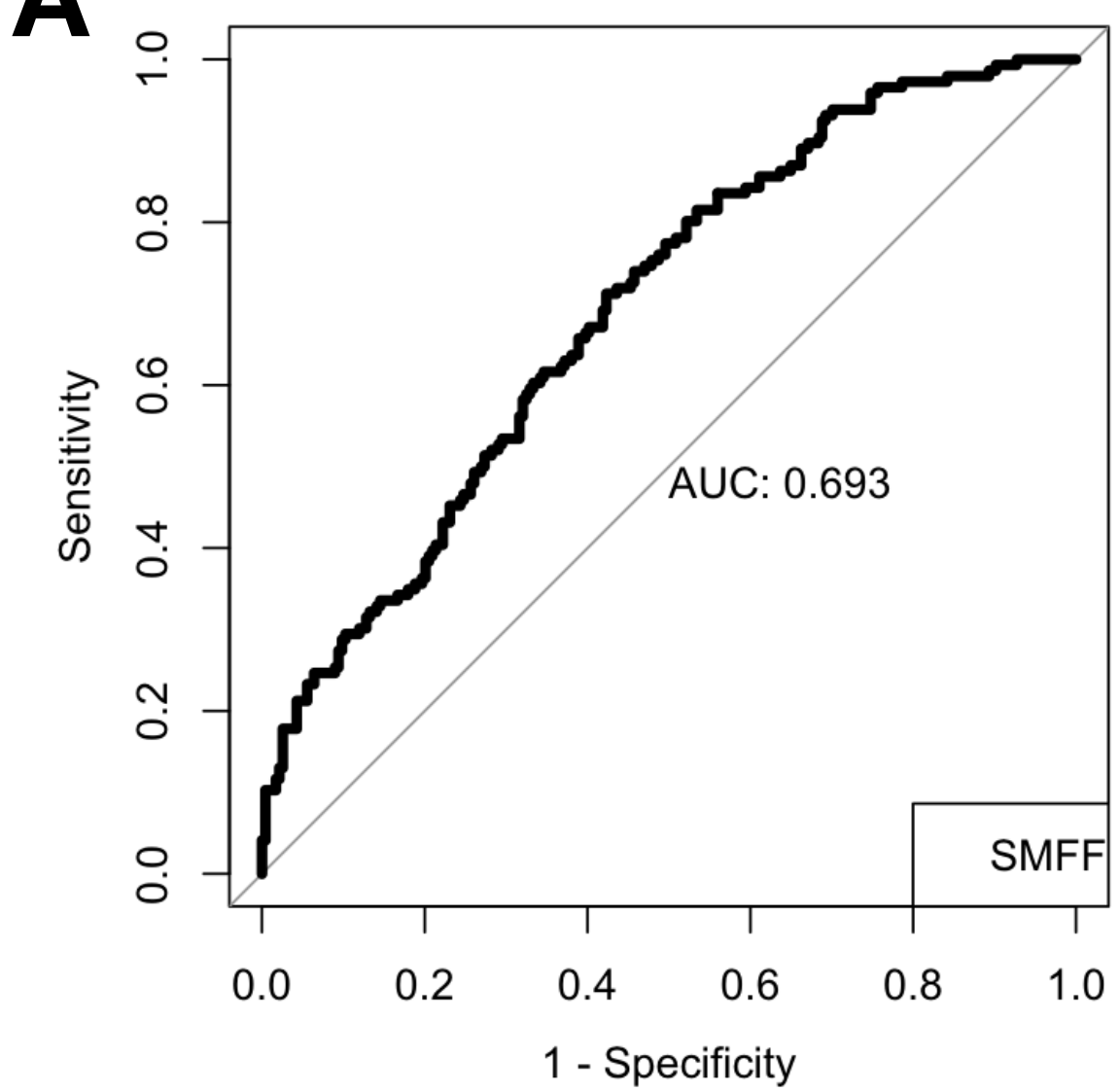**B**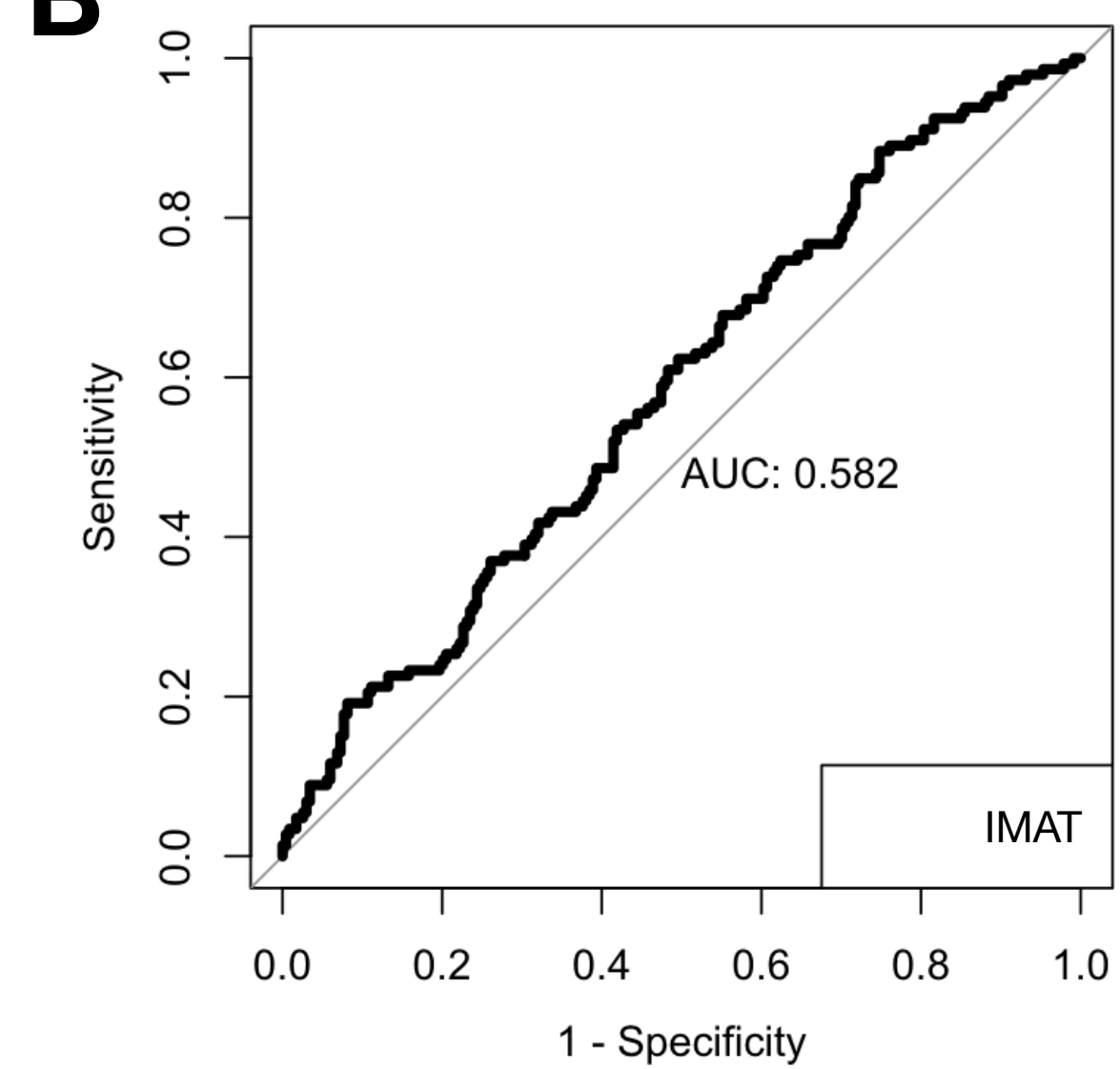**C**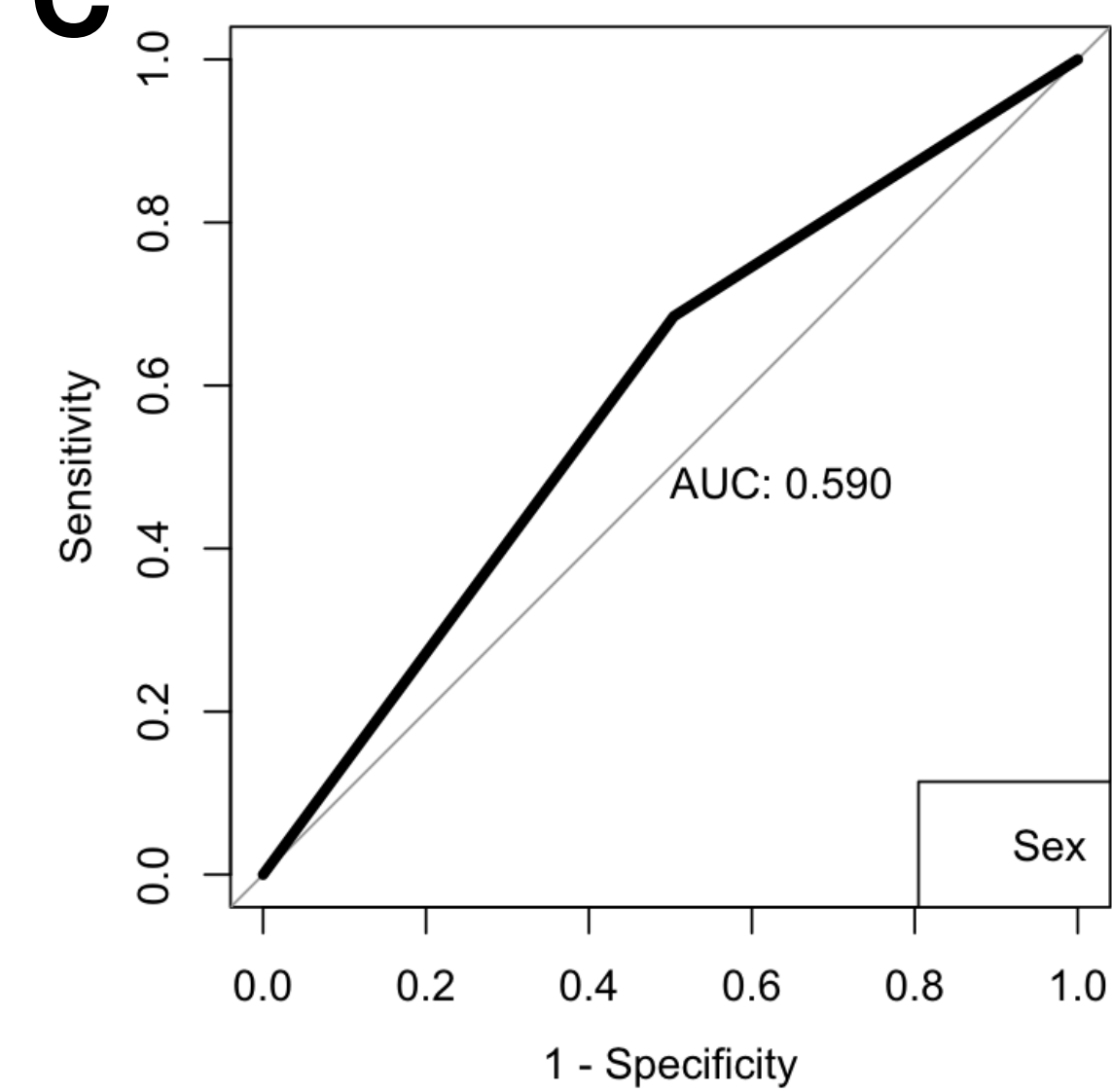**D**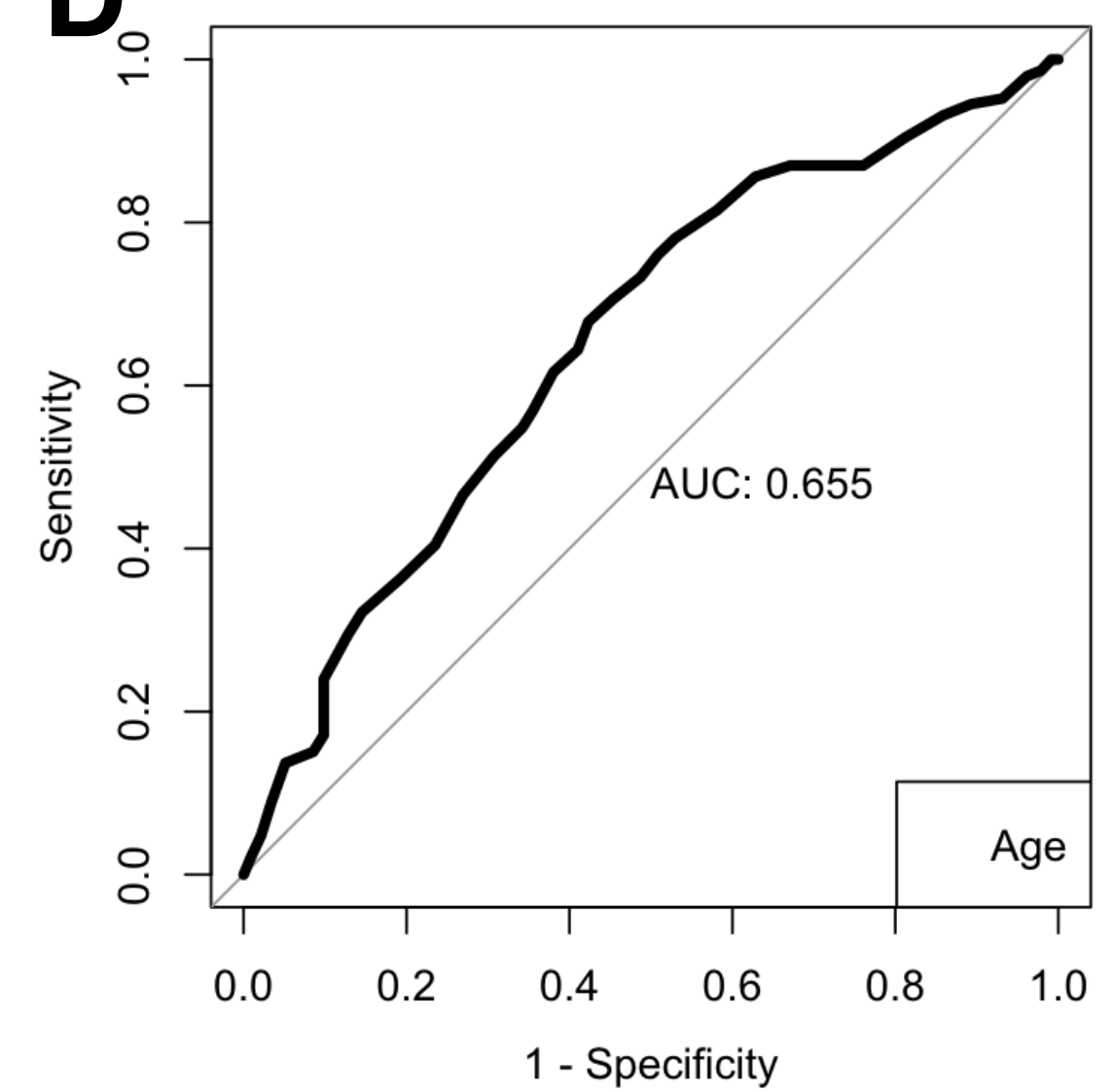**E**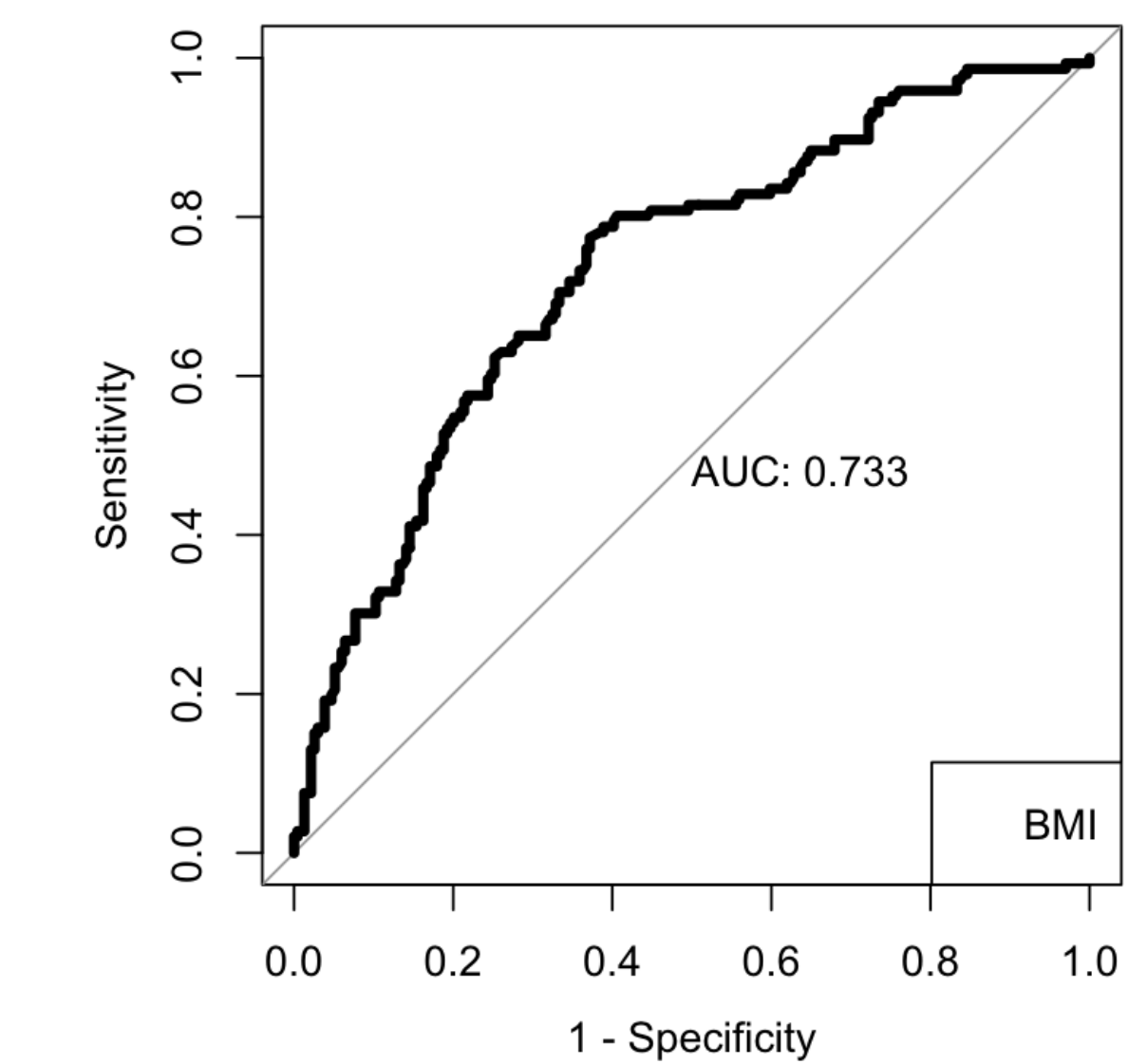**F**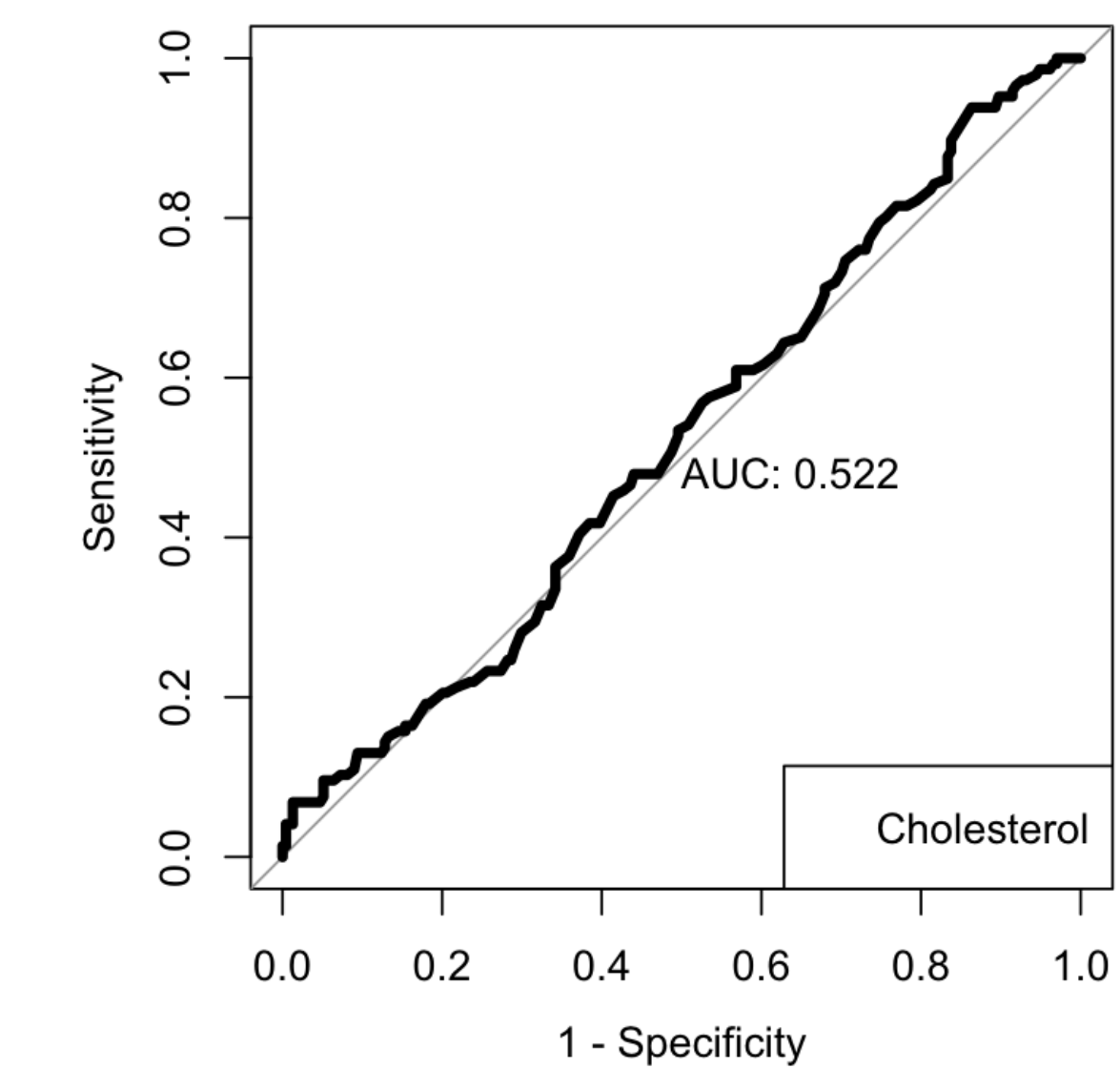**G**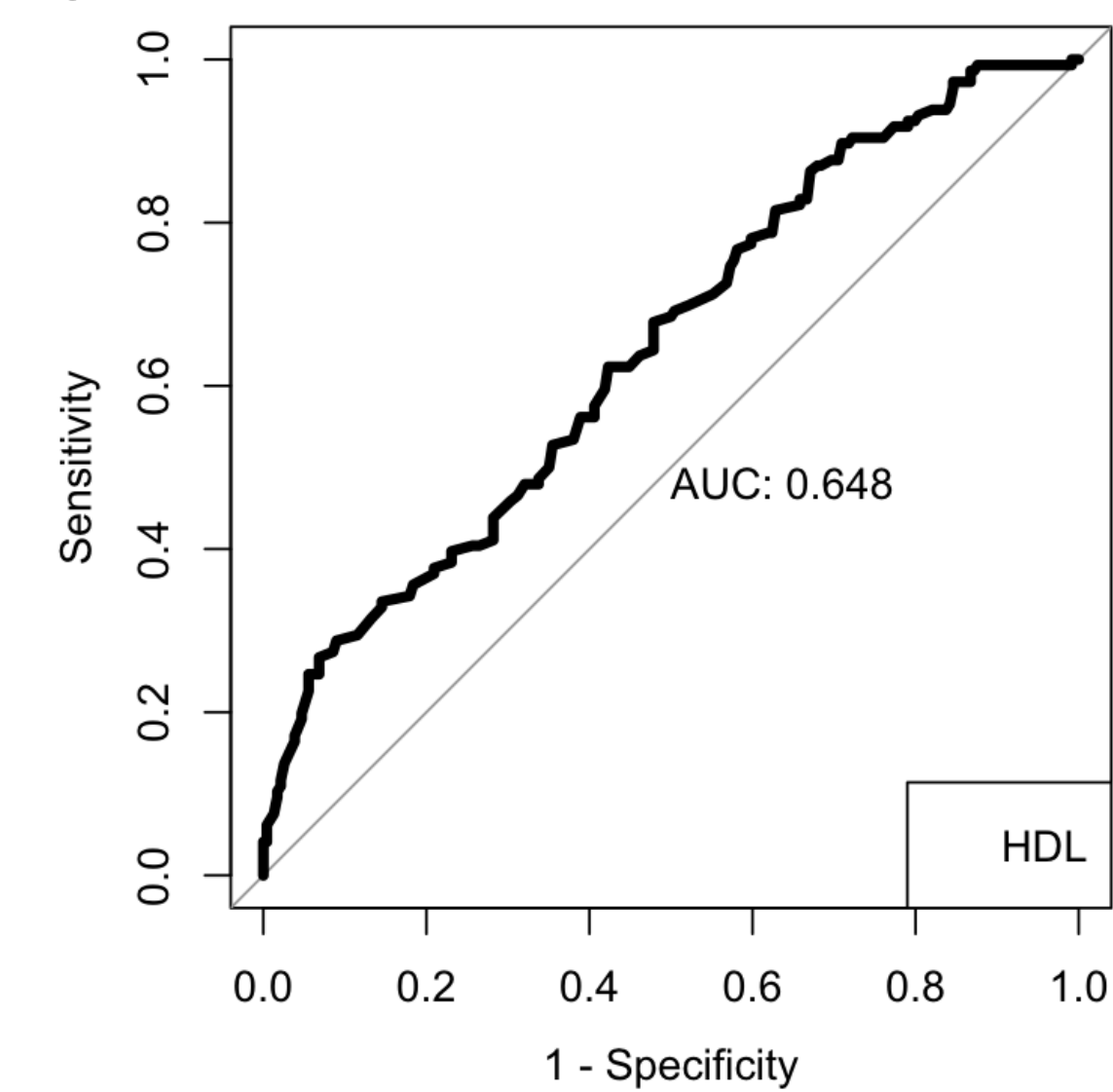**H**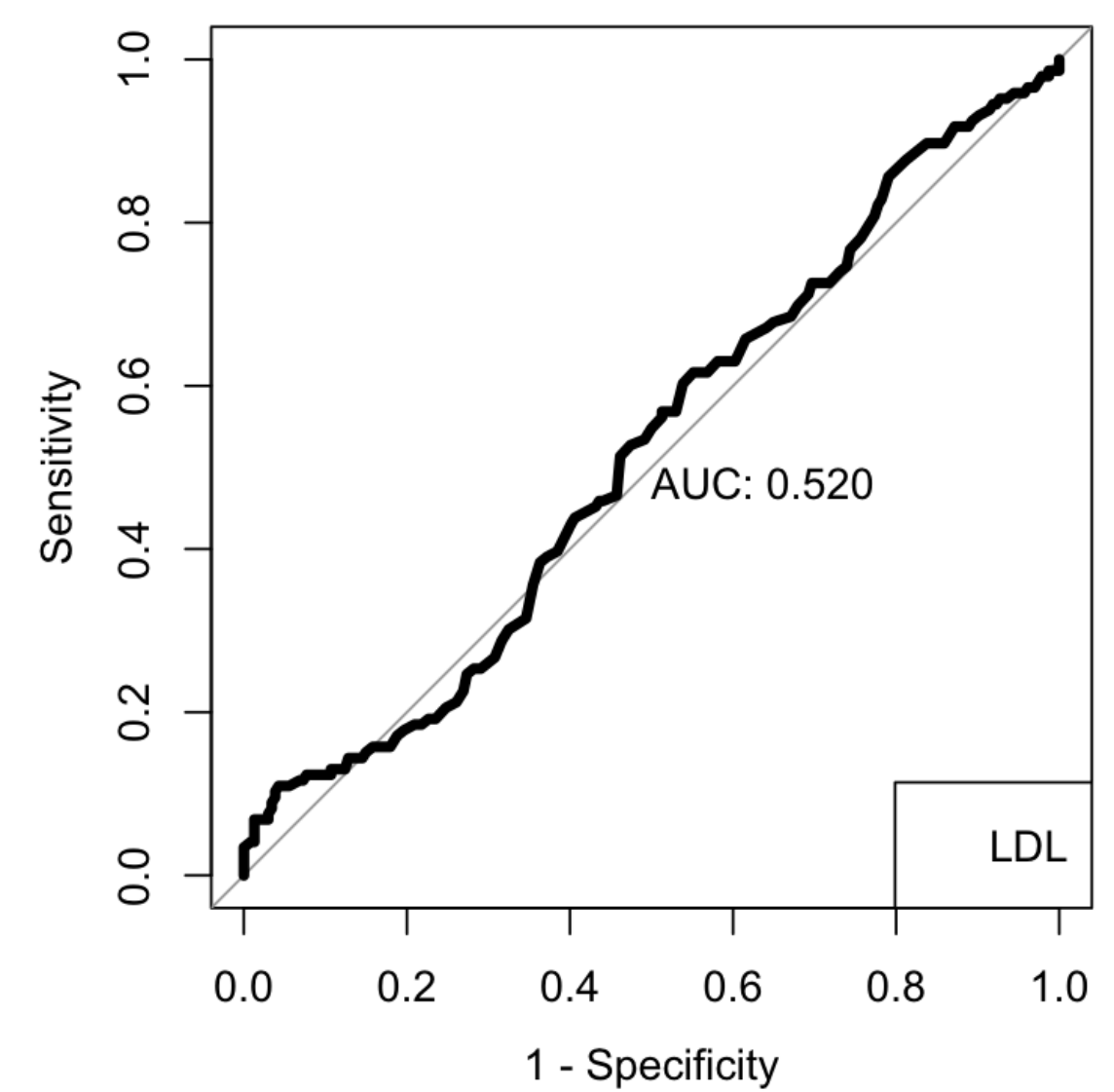**I**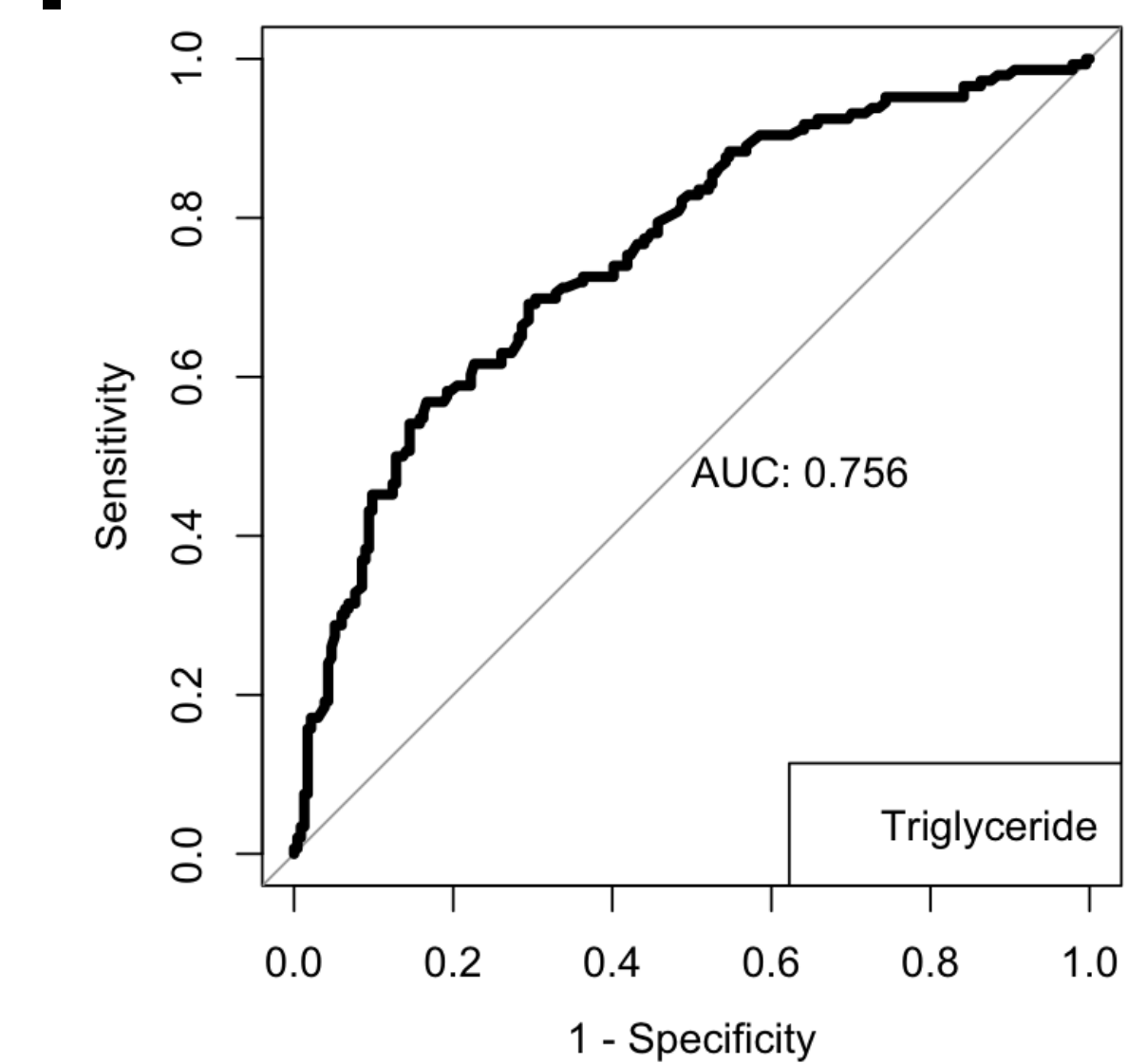**J**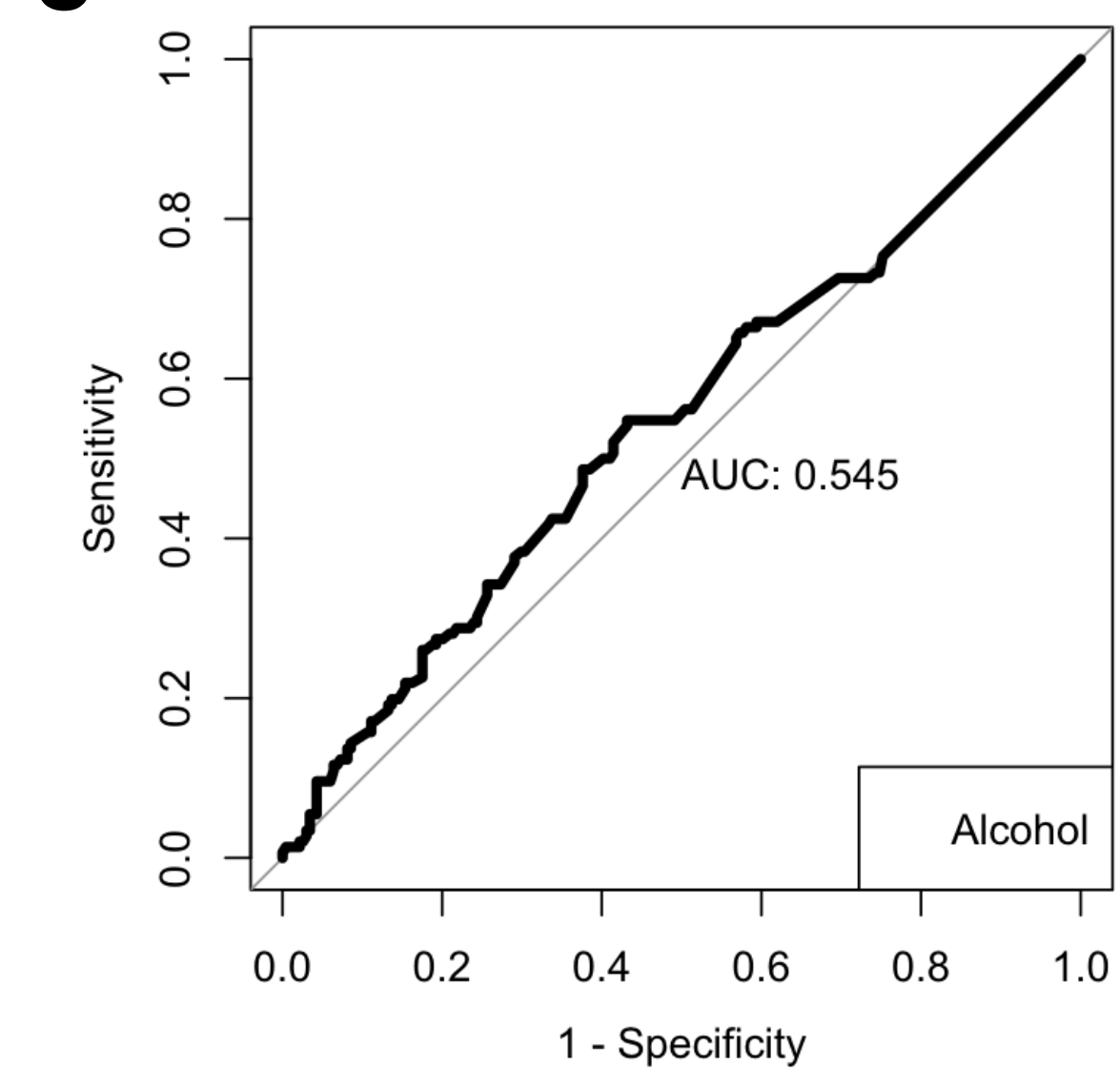**K**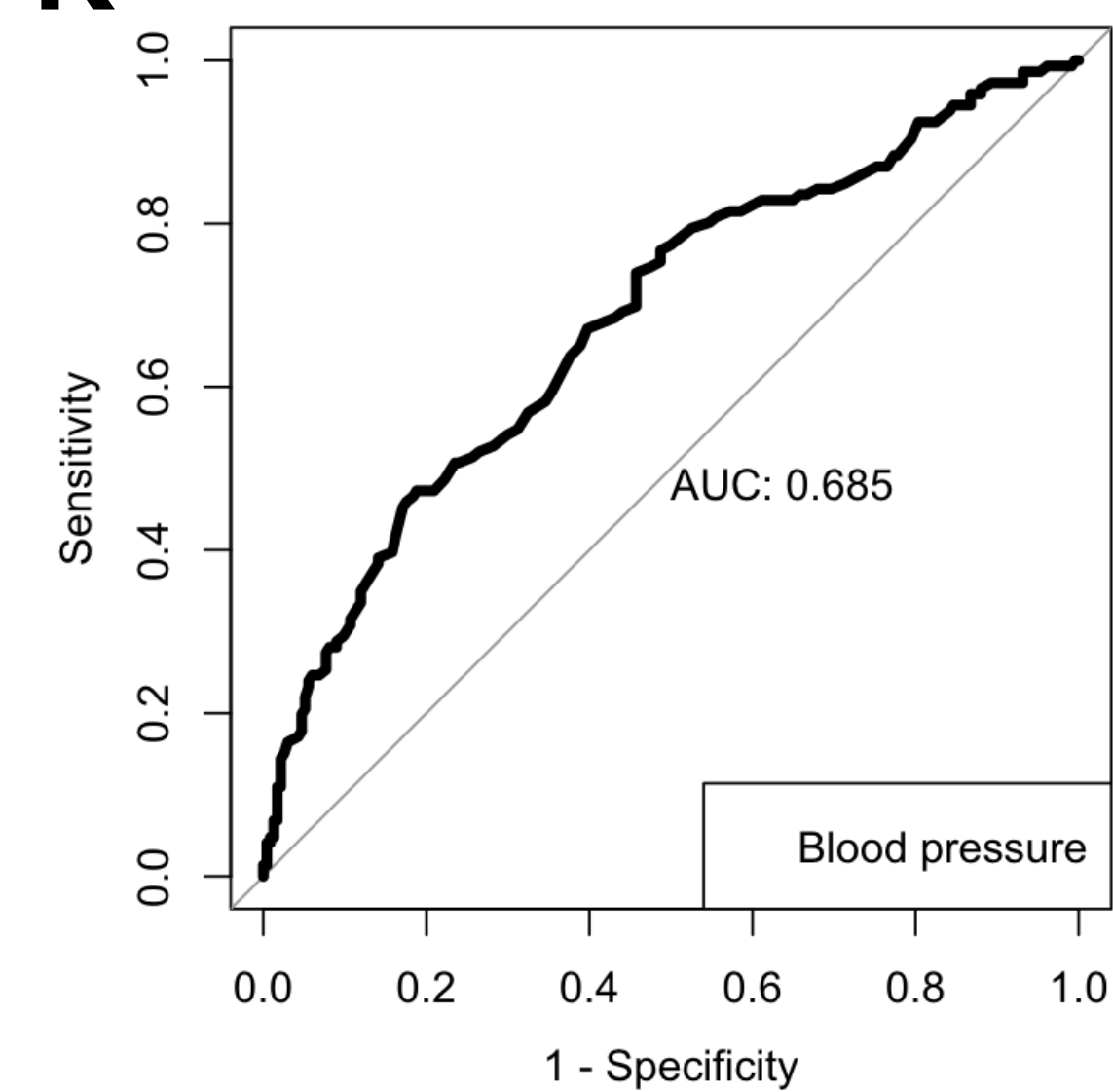**L**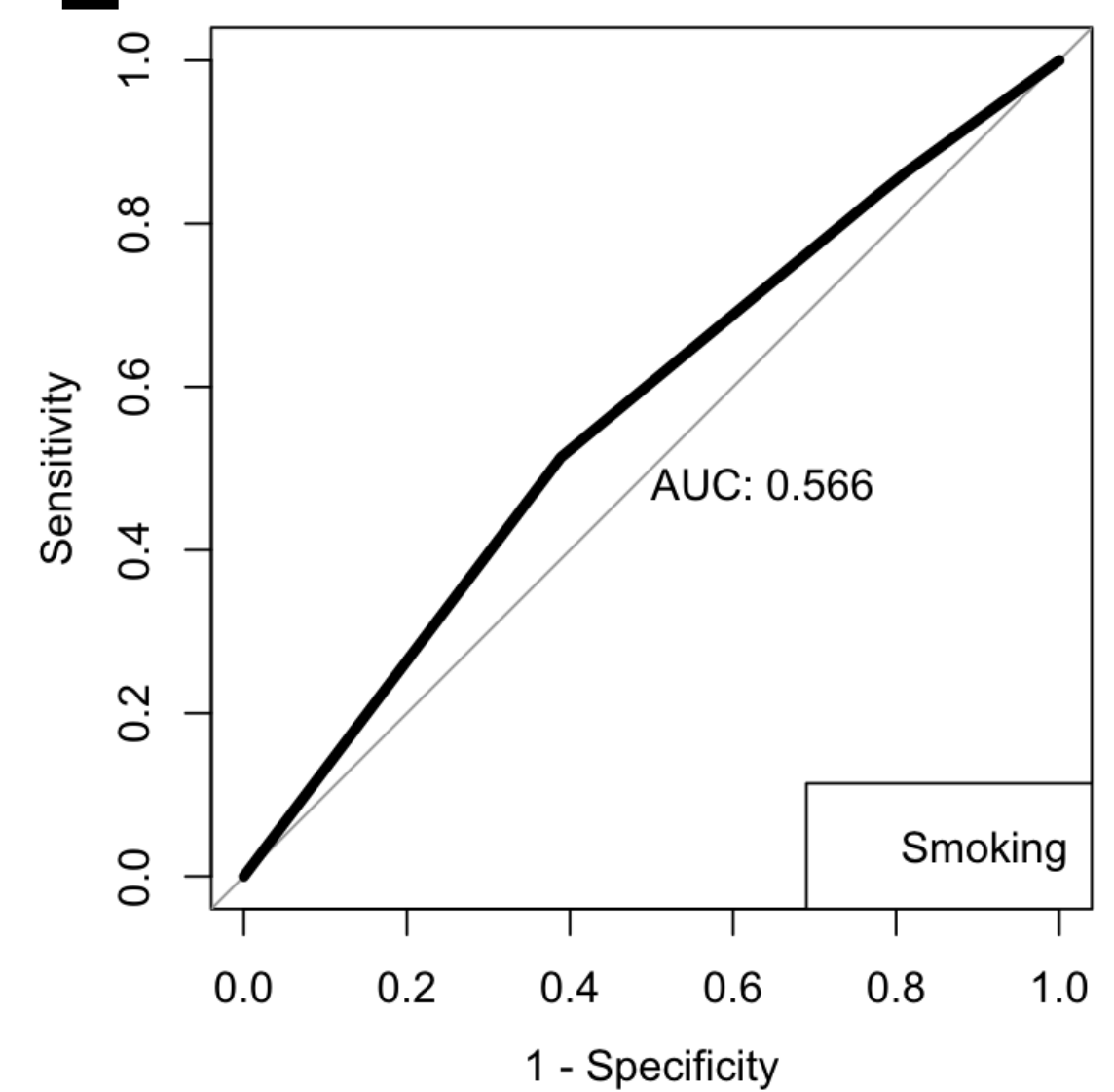

Supplement: Supplementary file 3 — Figure S3. Discrimination of baseline demographic and cardiometabolic risk factors for impaired glucose metabolism. Receiver operating characteristic curves and AUCs showing discrimination of the two myosteatosis measures (A) SMFF and (B) IMAT, baseline demographics (C) Sex, (D) Age, and (E) BMI, and cardiometabolic risk factors (F) total cholesterol, (G) HDL, (H) LDL, (I) triglycerides, (J) alcohol consumption, (K) systolic blood pressure, and (L) smoking status for impaired glucose metabolism. AUC, area under the curve. IMAT, intramuscular adipose tissue. SMFF, skeletal muscle fat fraction. [file JCSM-15-1750-s001.pdf]
